# Analyzing trend and forecasting of rainfall changes in India using non-parametrical and machine learning approaches

**DOI:** 10.1038/s41598-020-67228-7

**Published:** 2020-06-25

**Authors:** Bushra Praveen, Swapan Talukdar, Shahfahad, Susanta Mahato, Jayanta Mondal, Pritee Sharma, Abu Reza Md. Towfiqul Islam, Atiqur Rahman

**Affiliations:** 1 School of Humanities and Social Sciences, Indian Institute of Technology Indore, Simrol, Indore, 453552 India; 2 Department of Geography, University of Gour Banga, Malda, India; 3 Department of Geography, Faculty of Natural Sciences, Jamia Millia Islamia, New Delhi - 110 025, India; 4 Department of Disaster management, Begum Rokeya University, Rangpur, 5400 Bangladesh

**Keywords:** Climate sciences, Hydrology, Natural hazards

## Abstract

This study analyzes and forecasts the long-term Spatio-temporal changes in rainfall using the data from 1901 to 2015 across India at meteorological divisional level. The Pettitt test was employed to detect the abrupt change point in time frame, while the Mann-Kendall (MK) test and Sen’s Innovative trend analysis were performed to analyze the rainfall trend. The Artificial Neural Network-Multilayer Perceptron (ANN-MLP) was employed to forecast the upcoming 15 years rainfall across India. We mapped the rainfall trend pattern for whole country by using the geo-statistical technique like Kriging in ArcGIS environment. Results show that the most of the meteorological divisions exhibited significant negative trend of rainfall in annual and seasonal scales, except seven divisions during. Out of 17 divisions, 11 divisions recorded noteworthy rainfall declining trend for the monsoon season at 0.05% significance level, while the insignificant negative trend of rainfall was detected for the winter and pre-monsoon seasons. Furthermore, the significant negative trend (−8.5) was recorded for overall annual rainfall. Based on the findings of change detection, the most probable year of change detection was occurred primarily after 1960 for most of the meteorological stations. The increasing rainfall trend had observed during the period 1901–1950, while a significant decline rainfall was detected after 1951. The rainfall forecast for upcoming 15 years for all the meteorological divisions’ also exhibit a significant decline in the rainfall. The results derived from ECMWF ERA5 reanalysis data exhibit that increasing/decreasing precipitation convective rate, elevated low cloud cover and inadequate vertically integrated moisture divergence might have influenced on change of rainfall in India. Findings of the study have some implications in water resources management considering the limited availability of water resources and increase in the future water demand.

## Introduction

Rainfall is a key part of hydrological cycle and alteration of its pattern directly affect the water resources^1^. The changing pattern of rainfall in consequence of climate change is now concerning issues to water resource managers and hydrologists^2^. Srivastava *et al*.^3^ and Islam *et al*.^4^ reported that the changes of rainfall quantities and frequencies directly changing the stream flow pattern and its demand, spatiotemporal allocation of run-off, ground water reserves and soil moisture. Consequently, these changes showed the widespread consequences on the water resource, environment, terrestrial ecosystem, ocean, bio-diversity, agricultural and food security. The drought and flood like hazardous events can be occurred frequently because of the extreme changes of rainfall trend^5^. Gupta *et al*.^6^ documented that the amount of soil moisture for crop production is totally determined by the amount of rainfall. The monsoon rainfall plays a vital role for agriculture in India. 68% of cultivated land to the total cultivated land of India is occupying by the rain fed agriculture which supports 60% of livestock population and 40% of human population^7^. Hence, the research on the climate change or most specifically on the changes of rainfall occurrences and its allocation are the most significant way for sustainable water resource management. Therefore, the sustainable development of agriculture in India requires the noteworthy research on the identification and quantification of climate change^7^. Most importantly, a complete understanding of the precipitation pattern in the changing environment will help in better decision making and improve the adapting-capacity of the communities to sustain the extreme weather events.

Nowadays, the water resources have been considered as the key concern for any kinds of development program and planning which includes effective water resource management, food production sector and flood control. The uneven allocation of water supply throughout the country, because of the natural pattern of rainfall occurrence which varies significantly in space and time, is the main hindrance for the effective water resource management in India^8^. The climate change further accelerates this rainfall variability^7^. Consequently, many regions of the country receive huge amounts of rainfall during the monsoon, while others receive very less amount of rainfall and frequently experience the worst reality of water scarcity. Furthermore, the worst consequence of climate change, especially rainfall change, has been experiencing by the agricultural sector of this country. Because the rainfall has not been taking place when it is expected and vice versa^9^. Even, the high winds and hails have been frequently occurred. Consequently, enormous losses to crops have been taken place and the farmers who totally dependent on the agriculture have become devastated^7^. The freakish weather is causing havoc on the farmers community, as a results, the farmers have gone extreme to the committing of suicide. During the period 1995–2014, 300000 farmers have committed suicide in India^10^. Hence, it is noteworthy to evaluate whether there is any trend in rainfall and any pattern in variability^11^.

Therefore, to explore the variability and changes in pattern and existence of trend in rainfall over different spatial horizons have been the key aspects in the study of hydrology, climatology and meteorology worldwide^12–17^. In most studies, researchers have used parametric and non-parametric methods^18^ like regression test^19,20^, Mann-Kendall test^21,22^, Kendall rank correlation test^23^, Sen’s slope estimation^24,25^ and Spearman rank correlation test^26^. In the present study, the non-parametric test like Mann-Kendall test was applied to detect the trend in rainfall as it is one of the most often applied global methods for trend detection in hydrology, climatology and meteorology^27–31^. One of the major reason to use non-parametric tests in the present study is these can be used on independent time series data and are also not much sensitive to outliers^32^. However, the change detection analysis in the climatologic and hydrological data series is the important aspect for the trend analysis throughout the world^33,34^.The change detection methods include the Standard Normal Homogeneity Test (SNHT)^35^, Buishand range test^36^ and the Pettitt’s test^37^. The trend analysis was carried out before the change point analysis in many researches^32^. This approach can lead to misleading results as the information obtained from change point detection analysis has not been considered for the trend analysis^38^. Hence, Li *et al*.^36^ highly recommended performing the change detection analysis first followed by the trend analysis. The results obtained from this approach are more reasonable and reliable.

In India, several attempts have already been done in the past to detect rainfall trends in regional and national levels^39–46^. The research on trend analysis has believed to be a useful tool as it provides the significant information about the possibility of the future changes^4,47,48^. However, multiple studies have conducted trend analysis using MK test and reported that India as a whole has not recorded significant trend of increase or decrease in annual average rainfall^7,46,49^. Many researchers identified significant trend in long-term rainfall in India^2,44,50–53^, while the significant long-term trend has not been detected in monsoon rainfall on a national scale^7^. Patra *et al*.^54^ studied the trend analysis in monthly, seasonal and annual rainfall over Orissa and reported that long-term insignificant negative trend was detected in annual and monsoonal rainfall, while the positive trend was detected in post- monsoon season. But to the best of authors knowledge, most of the researchers applied non-parametric tests to detect and followed by trend analysis. Therefore, to get reliable and reasonable results of trend, we have obtained both approaches like trend analysis before change point and first change point analysis followed by trend detection.

Sen^55^ developed the innovative trend analysis that has been utilized for detecting trend in meteorological, hydrological and environmental variables^56–60^. The innovative trend analysis has the worldwide applicability over the non-parametric approach^61^ like Mann-Kendal test, spearman’s correlation test and Sen’s slope estimation because these non-parametric test are very much sensitive to the distribution assumptions, serial correlation, size of the time series data and seasonal cycle^62^. Von Storch^63^ stated that if the data series have statistically significant serial correlation, the MK test can generate no noteworthy trend existence in the data series. Sen^55^ stated that the effective, efficient and optimum water resource management needs to identify the trend not only monotonically over time, but also needs to identify trends separately which have the high, medium and low value which can be possible to identify by the innovative trend. In addition, the innovative trend is not sensitive to serial correlation, non-linearity, and size of the time series data and gives the robust and powerful result with less error. Therefore, researchers prefer the innovative trend analysis for detecting trend in hydrological, meteorological studies^18,44,55,64,65^. Therefore, in the present study, apart from Mann-Kendall test, the innovative trend was also utilized for detecting trend. In India, very few studies were conducted to detect trend using innovative trend analysis. But to the best of authors’ knowledge, the application of innovative trend to detect seasonal rainfall for whole India would be the first application.

As we have considered detecting change point using MK test before change point analysis, MK test after change point analysis and innovative trend analysis, the planners of water resource management, environmentalist, and hydrologists can have the chance to compare the result of all these techniques and get high precision trend information in annual and seasonal rainfall. Therefore, in this line of thinking, we can conclude that the study has the quite novelty.

The trend analysis facilitates to understand the present and past climatic changes, but the future forecasting is more useful for the planners to execute the proper planning taking into account future changes in climatic variables^66^. To project future information of the climatic variables, in addition to the complicated climate model which works on global scale, regional forecasting could be carried using statistical techniques and machine learning soft computing techniques^67,68^. To execute the physical models, large numbers of database, high configured system, advanced technology, technical expert are required. The physical models are both time and money consuming, although they provide highly reliable results. The statistical techniques like auto regressive (AR), moving average (MA), auto regressive moving average (ARMA) and auto regressive integrated moving average (ARIMA) have several limitations such as AR model regresses past values, while MA model utilizes past error as the explanatory variables and ARMA model can perform for stationary time series data^67^. However, recently, the application of artificial intelligence (AI) models like machine learning techniques have gained attention^69^. Unlike physical models, the AI models perform very well as it does not require huge information, but can handle huge and complicated data sets if provided^70^. It can perform in non-stationary time series data^71^. Darji *et al*.^67^ conducted review survey regarding the rainfall forecasting using neural networks and reported that back propagation based neural network performs well to forecast rainfall. In the present study, we utilized multilayer perceptron (MLP) algorithm based neural network for predicting the rainfall. The MLP based ANN works using back propagation algorithm. However, the application of MLP based artificial neural network (ANN) for predicting and forecasting of multifaceted hydrological and climatic phenomena and has gained popularity across the globe as it provides reliable results^72–81^. The novelty of the present study is that the application of MLP-ANN is not concentrated on the one or more states or overall India rather the present study considers predicting and forecasting rainfall for thirty-four meteorological sub-divisions of India. Therefore, the study will be highly beneficial as it worked on the whole India in micro level.

Therefore, based on the previous literatures, research gaps, the objectives of the present study are: (1) to analyze the variability and trend analysis in the overall annual and seasonal rainfall for thirty-four meteorological sub-divisions; (2) to detect the change point for all sub-divisions; (3) to explore the change point wise rainfall variability and trend analysis; (4) to detect trend using innovative trend analysis; (5) to predict and forecast rainfall up to 2030 for all sub-divisions; (6) to investigate the causes of the changes of rainfall pattern.

## Material and Methods

### Study Area and data source

The geographical location of India is between 8.4° and 37.6°N latitude and 68.7° and 97.25°E longitude. The whole India can be categorized into four homogenous climatic regions such as North-West India (NW India), North-East India (NE India), Central India and Peninsular India on the basis of the distribution and occurrence pattern of rainfall and temperature^46^. Although the whole country experiences almost all types of climate due to its physiographic location^59^. However, India observes the four seasons, like summer season (January-February), winter (March-May), monsoon (June-September) and post-monsoon (October-December)^82^. The India receives 117 cm rainfall, out of which 80% of rainfall is observed during monsoon month.

**Figure 1:**
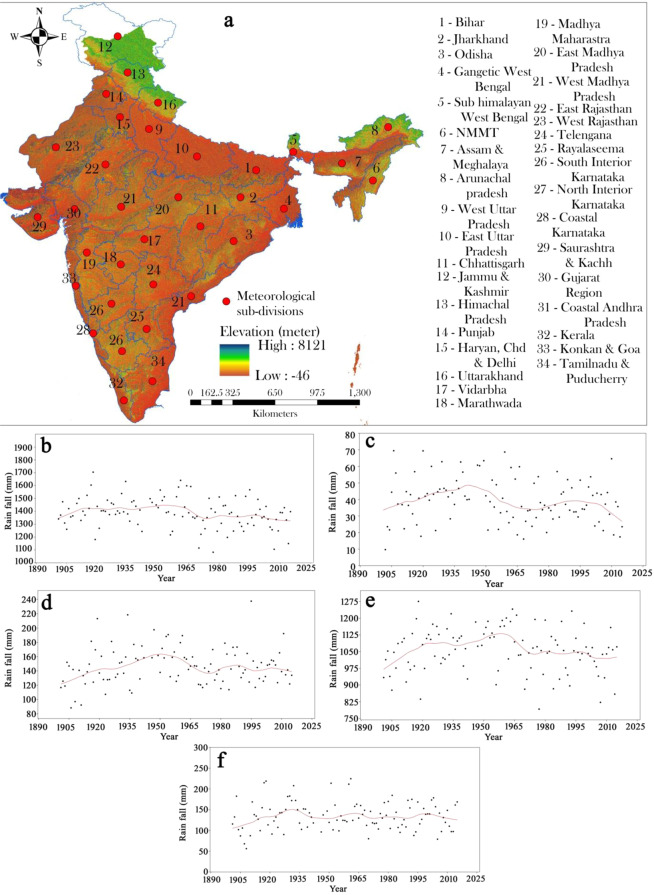
(**a**) Geographical location of the study area; LOWESS curve on annual and seasonal rainfall where figure shows (**b**) annual rainfall, (**c**) winter (**d**) summer (**e**) monsoon and (**f**) post monsoon rainfall.

The whole Indian region has been sub-divided into thirty-four meteorological subdivisions by the Indian Meteorological Department (IMD) where the climatic parameters like rainfall, temperature, wind speed, humidity have been recorded since 1901 (Fig. 1a). In this study, we obtained rainfall data for 115 years (1901–2015) from thirty-four meteorological sub-divisions. The collected data was continuous in nature with no missing values.

#### Method for trend analysis

In this paper we used the Mann-Kendal test^18^, a non-parametric statistical test based on rank system, to detect the trend in long-term rainfall data series. The MK test is mainly used for detecting trend in hydro-climatic data series as the lower sensitivity to any sudden change^83,84^. To perform this test, it is essential to evaluate the presence of serial correlation within the data series^85^. A positive serial correlation can support the expected number of bogus positive products in the MK test^86^. For this reason, the serial correlation must be excluded prior to applying the MK test. To eliminate the serial correlation, the trend free pre-whitening (TFPW) technique proposed by Yue and Wang^85^ was used.

The MK test was calculated using Eq. 1:

1 $$S=\mathop{\sum }\limits_{i=1}^{n}\mathop{\sum }\limits_{j=i+1}^{n}\mathrm{sgn}({K}_{j}-{K}_{i})$$

Where,

$${\rm{sgn}}({K}_{j}-{K}_{i})=(\begin{array}{ccc}1 & if({K}_{j}-{K}_{i}) & \rangle 0\\ 0 & if({K}_{j}-{K}_{i}) & =0\\ -1 & if({K}_{j}-{K}_{i}) & \langle 0\end{array})$$

In a time series, *K*_*i*_*, i* = *1, 2, 3, ……….n*, the value of S is supposed to be similar as the normal distribution with a mean 0 and while the discrepancy of statistics S has been computed using Eq. 2:

2 $${\rm{var}}(s)=\left[\frac{n(n-1)(2n+5)-\mathop{\sum }\limits_{y=1}^{x}{t}_{y}({t}_{y}-1)(2{t}_{y}+5)}{18}\right]$$

The *Z*_*MK*_ value is used to find out that the time series information is demonstrating a significant trend or not. The *Z*_*MK*_ value is computed using Eq. 3:

3 $${Z}_{MK}=\left(\begin{array}{ccc}\frac{S-1}{\sqrt{{\rm{var}}(S)}} & if & S\rangle 0\\ 0 & if & S=0\\ \frac{S+1}{\sqrt{{\rm{var}}(S)}} & if & S\langle 0\end{array}\right)$$

The positive and negative values of Z in a normalized test statistic reflect the increasing and decreasing trend, respectively, while the Z having 0 values reflects a normal distributed data series.

#### Method for change point detection

In the present study, we utilized Pettitt test proposed by Pettitt^37^ and Standard Normal Homogeneity test (SNHT) developed by Alexandersson and Moberg^35^ to explore the presence of the abruptly shifting change points in the time series annual and seasonal rainfall dataset for all meteorological sub-divisions.

#### Pettitt Test

The Pettitt test is a distribution-free rank based test, used to discover noteworthy changes in the mean of the time series. It is more helpful when the hypothesis testing about location of a change point is not necessary. This test has been used extensively to identify the changes observed in climatic and hydrological data series^87,88^. When length of a time series is represented by *t* and the shift take place at m years, the consequential test statistics are expressed as given in Eq. (4).The statistic is similar to the Mann- Whitney statistic, which characterized by two samples, such as k1, k2…, km and km+1, k2…, kn:

4 $${U}_{t,m}=\mathop{\sum }\limits_{i=1}^{m}\mathop{\sum }\limits_{j=t+1}^{t}{\rm{sgn}}({K}_{i}-{K}_{j})$$

where $\mathrm{sgn}$ in Eq. 4 is defined by Eq. 5:

5 $${\rm{sgn}}({K}_{i}-{K}_{j})=(\begin{array}{ccc}1 & if({K}_{i}-{K}_{j}) & \rangle 1\\ 0 & if({K}_{i}-{K}_{j}) & =0\\ -1 & if({K}_{i}-{K}_{j}) & \langle 1\end{array})$$

The test statistic *Ut,m* is calculated from all haphazard variables from 1 to *n*. The majority of distinctive change points are recognized at the point where the magnitude of the test statistic |*Ut,m* | is highest (Eq. 6).

6 $${Z}_{T}=Ma{x}_{1\le t\langle m}|{U}_{t.m}|$$

The probability of shifting year is estimated when |*Ut,m* | is maximum following Eq. 7:

7 $$P=1-\exp \left(\frac{-6{Z}_{T}^{2}}{{K}^{2}+{K}^{3}}\right)$$

If the *p-*value is less than the significance level α, the null hypothesis is considered to be rejected.

#### Standard Normal Homogeneity Test (SNHT)

The standard normal homogeneity test is also known as the Alexanderson test. This test is applied to detect sudden shift or presence of change point in time series of climatic and hydrologic datasets. The change point has been detected following Eq. 8:

8 $${T}_{s}=\,{\rm{\max }}\,{T}_{m},1\le m < n$$

The change point refers to the point, when T_s_ attains maximum value in the data series. The T_m_ is derived using Eq. 9:

9 $${T}_{m}=\overline{m}{z}_{1}+(n-m){\overline{z}}_{1},m=1,2,\mathrm{...}.,n$$

Where,

10 $${\bar{z}}_{1}=\frac{1}{m}\mathop{\sum }\limits_{i=1}^{n}\frac{({M}_{i}-\overline{M})}{s}$$

where $\bar{m}$ represents the mean and *s* represents the standard deviation of the sample data.

#### Buishand Rang Test

The Buishand range test is also called as Cumulative Deviation test, which is calculated based on the adjusted biased sums or cumulative deviation from mean. The change point using this test is detected following Eqs. 11 and 12:

11 $${R}_{0}^{\ast }=0\,and\,{{\rm{R}}}_{m}^{\ast }=\mathop{\sum }\limits_{t=1}^{m}{P}_{t}-{P}_{mean})$$

Where,

$$\begin{array}{c}m=1,\mathrm{2.........}.,n\\ {R}_{m}^{\ast \ast }={{\rm{R}}}_{m}^{\ast }/\sigma \end{array}$$

12 $$S=Max|{R}_{m}^{\ast \ast }|-Min|{R}_{m}^{\ast \ast }|,0\le m\le n$$

The $S/\sqrt{n}$ is then estimated using the critical values proposed by Buishand^38^.

### Methods for innovative trend analysis

The innovative trend analysis, proposed by Şen^55^, was applied to detect the trend in long-term time series rainfall data of India. The innovative trend analysis has greatest advantages over the MK test and other parametric and non-parametric statistical tests, which is that it does not need any assumptions like non-linearity, serial correlation and sample numbers. However, the graph of this test is being plotted on a Cartesian coordinate system depending on a sub-section time series. As per the method’s requirement, the time series rainfall data has been sub-divided in two time series data sets: 1901–1957 and 1958–2015. Both segments were fixed in an ascending order. Typically the first sub-series (x_i_) was represented on x axis, while the other sub-series (y_i_) was represented on y axis. The data is plotted on the 1: 1 line. If the data is plotted along the 1:1 line, it indicates simply no trend within the time series. If the data is being plotted above the 1:1 line, it will state that the moment series shows an increasing trend, and if the data falls under the 1:1 line, it will indicate the negative trend^56,58^. If the scatter plot is more close to the 1: 1 path, the trend alter slope of that time period series will be weaker, along with the farther far from the 1:1 tier it is, the particular stronger the excitement change incline in the time series^55^. Often the straight-line trend slope displayed by the ITA method can be expressed as by the Eq. 13^56^:

13 $$s=\frac{1}{n}{\sum }_{i=1}^{n}\frac{10(\bar{y}-\bar{x})}{n}$$

Where s is the indicator of trend having the positive values that represent the increasing trend, while the negative values represent the decreasing trend. The $\bar{{\rm{y}}}$ and $\bar{{\rm{x}}}$ are the arithmetic average of the first sub-series (xi) and second sub-series (yi) respectively. Furthermore, n is represented by the number of collected data products. The indicator is then multiplied by 10 for comparing with MK test^61^.

### Method for analyzing rainfall changes

To calculate atmospheric oscillations on rainfall trend variation, winter and summer precipitations and moisture divergence during 1979–2015 on 1.25° × 1.25° grids were obtained from the European Centre for Medium Range Weather Forecasts (ECMWF), ERA-5 (http://apps.ecmwf.int/datasets/data/interim-full-daily). The ERA5-Interim is the most recent ocean-atmospheric changes reanalysis datasets available since 1979 forwards. In addition, low cloud cover dataset was also derived from the ECMWF ERA5 data to assess the effect of cloud cover on rainfall variation^89^. We have quantified the influence of atmospheric circulation changes on the trend patterns in rainfall. At first, we have detected a recent significant change point of annual mean rainfall based on Pettit test for the period 1979–2015 in India and observed that the mean rainfall has a change point after 2000. Then, the change in circulation of the two periods before and afterword the changes are quantified by subtracting 1979–2000 from 2001 to 2015using the ECMWF ERA5 reanalysis data. The GrAdS software (http://cola.gmu.edu/grads/) was used to prepare the spatial maps.

### Method for future forecasting

Several probabilistic and deterministic methods like ARMA, ARIMA, SARIMA are usually employed to predict the hydrological and climatic datasets. These techniques have many drawbacks like serial correlation, non-linearity, and biasness to predict the non-linear hydro-climatic data sets. Therefore, the newly developed artificial intelligence (AI) models can able to overcome this drawback. Hence, the application of AI models is now widely popular to solve the environmental problems. However, in the present study, we employed artificial neural network, a popular AI model, to predict and forecast the rainfall of thirty-four meteorological sub-divisions. The ANN is a non-linear black-box AI model. This model works like parallel-distributed information processing system which reflecting biological structure of brain as it comprised of simple neurons and links that process information to establish an association between the inputs and outputs. Likewise the function of brain, the ANN model is working using feed-forward multilayer perceptron algorithm. This structure is consisting of three layer, such as an input layer, one or more hidden layer and an output layer. We selected the number of neurons in the hidden layer by trial and error procedure. The ANN learning is based on the structure and functions of biological neural networks i.e. enclosing adjustment to the synaptic links that present in the core of the neurons. The multi-layer perception (MLP) is one of the most extensively employed neural network typologies. The MLP with two hidden layers is the most used classifier for the stationary pattern classification. MLPs are generally functioned using the backpropagation algorithm. The back-propagation principle flourishes the errors from the network and permits adjustment of the unseen units. Two crucial characteristics of multilayer perceptron are:

(1) The non-linear processing elements (PEs) having non-linearity that should be smooth, so that the logistic functions and hyperbolic tangent can be frequently employed; and

(2) Their enormous interconnectivity (any constituent of a given layer feeds all the constituents of the next layer).

In the present study, we divided the whole rainfall datasets into training and testing datasets. Then we normalized the data and applied ANN model for predicting the annual rainfall. We applied ANN model again and again on the same rainfall datasets by changing the model’s parameters like seed, momentum, learning rate until the best ANN model achieved for prediction rainfall. In addition, we evaluated the performance of ANN for predicting using Root Mean Square Error (RMSE) techniques. When we achieved the best ANN model, we fixed the model parameters and applied on the rainfall data to forecast rainfall up to 2030. However, the rainfall prediction and forecasting were performed in Weka software (version 3.9) (https://weka.informer.com/3.9/) and the mappings were done in Arc GIS (version 10.3) software.

#### Method for change rate calculation in rainfall time series data

The simple statistical method, percentage change is applied to calculate the change rate of the annual and seasonal rainfall for pre change and post change point. This method is very simple but the function of this method is much effective. It is calculated using Eq. 14.

14 $$Percentage\,change( \% )\,over\,period=\left(\frac{Average\,rainfall\,of\,post\,change\,point-Average\,rainfall\,of\,pre\,change\,point}{Average\,rainfall\,of\,pre\,change\,point}\right)\ast 100$$

#### Spatial mapping using Kriging interpolation method

In this study, the ordinary kriging method was employed to interpolate the statistical data and prepare the spatial rainfall map. The ordinary kriging method works on the data having statistical properties or spatial autocorrelation and this method employed the semi-variogram model to represent the spatial linkage (autocorrelation). The semi-variogram model evaluates the power of spatial association as a function of distance between the data. Kriging is used to estimate the values Z*(*x*_0_) at the point *x*_0_ expressed^90^ in Eq. 15 and the estimation of error variance ${\sigma }_{k}^{2}({x}_{0})$, expressed in Eq. 16.

15 $$Z\ast ({x}_{0})=\mathop{\sum }\limits_{i=1}^{n}{\lambda }_{i}z({x}_{i})$$

16 $${\sigma }_{k}^{2}({x}_{0})=\mu +\mathop{\sum }\limits_{i=1}^{n}{\lambda }_{i}\gamma ({x}_{0}-x{}_{i})$$

Where *λ*_*i*_ refers to weights; *μ* refers to LaGrange constant; and (*x*_0_ − *x*_*i*_) represents the semi-variogram value equivalent to the distance between *x*_0_ and *x*_*i*_^91,92^. Nielsen and Wendroth^93^ suggested that a semi-variogram is comprised of the regionalized variable theory and intrinsic hypotheses and it is expressed as follows:

17 $$\gamma (h)=\frac{1}{2N(h)}{\mathop{\sum }\limits_{i=1}^{N(h)}[Z({x}_{i})-Z({x}_{i}+h)]}^{2}$$

Where, *γ*(*h*) represents semi-variance, h represent lag distance, Z refers to the rainfall-related parameters, N(h) is the number of couples of locations divided by the lag distance h, $Z({x}_{i})$, and $Z({x}_{i}+h)$ refers to the values of Z at the positions *x*_*i*_ and *x*_*i*_ + *h*^94^. The empirical model of semi-variogram generated from collected data which was fitted to the theoretical semi-variogram. It was utilized to create geo-statistical properties which further include nugget structured and the sill variance as well as the distance parameter. In addition, the nugget-sill ratio has been calculated in order to characterize the spatial dependency of the statistical values. A nugget-sill ratio of 75% reflects a weak spatial dependency; or else the dependency is considered moderate.

Furthermore, all mappings in the present work were performed using kriging interpolation method in Arc GIS (version 10.3) software.

## Results and Discussion

### Descriptive Analysis of annual rainfall

We calculated the descriptive statistics of the annual rainfall since 1901 to 2015 for thirty-four meteorological sub-divisions of India. Results show that the South Indian meteorological divisions i.e. Kerala, Tamil Nadu, and Konkan & Goa have observed the highest average rainfall (3396.64 mm. 2930 mm. and 2974 mm. respectively). While, the minimum average rainfall has been recorded in the sub-divisions of West India meteorological divisions i.e. West Rajasthan (288.74 mm.), Saurashtra and Kutch (494.27 mm.), Haryana (535.47 mm.), Delhi and Chandigarh (596.16 mm.). The standard deviation of rainfall for whole India varies from 1242.04 to 108.99 mm. The highest variation (standard deviation) in rainfall was observed in Arunachal Pradesh meteorological sub-division, followed by Coastal Karnataka (480.98 mm.), Konkan & Goa (478. 49 mm.), while the minimum variation was recorded in Western Rajasthan (108. 99 mm., followed by North Interior Karnataka (135.33 mm), Haryana Delhi and Chandigarh (142. 64 mm). The skewness of the rainfall ranges from −0.81 to 1.05 for all sub-divisions of India. The negative skewness was found in Tamil Nadu (−0.81), Bihar (−0.39), Konkan & Goa (−0.21), Jharkhand (−0.15), West Uttar Pradesh (−0.09), East Madhya Pradesh (−0.02) and Madhya Maharashtra (−0.001). While rest of the meteorological sub-divisions were observed positive skewness.

The arithmetic mean is not significant (robust) to local variations^95^. Therefore, LOWESS regression curve was applied on the seasonal rainfall to minimize the local variation. A cluster of researchers used this statistical method and achieved satisfactory findings over the arithmetic mean^96,97^. The findings of LOWESS curve indicate an increasing pattern of annual rainfall upto1965, while a decreasing pattern of rainfall was found after the year 1970. (Fig. 1b). The findings of LOWESS curve for the winter season (Fig. 1c) showed that the increasing rainfall pattern was observed for the periods of 1935–1955 and 1980–1998. A sudden decrease in the trend was observed after 1955 and 1998. In the case of summer and monsoon season (Fig. 1d,e), the curve indicates that the negative trend was observed after the 1960. The identical result was found in the work of^98^. Whilst, in the case of post-monsoon (Fig. 1f), the LOWESS regression curve indicates the positive trend was found during 1925–1935 and 1955–1965. The negative trend for post monsoon was observed after 1995.

### Long Term Pattern and variation of average annual and seasonal rainfall

**Figure 2:**
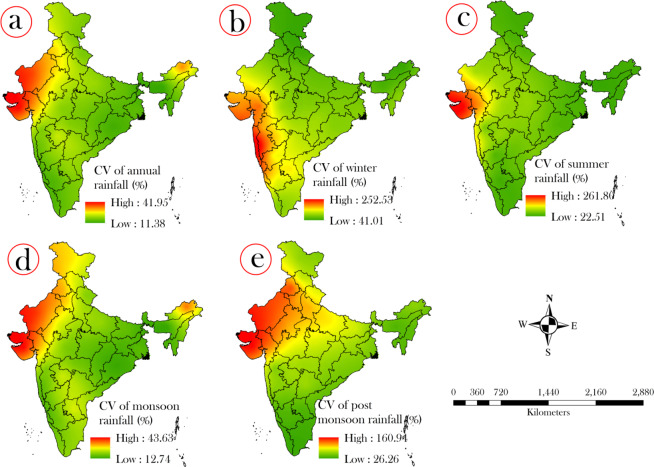
Meteorological subdivision wise spatial variations using the coefficient of variation (CV) in annual and seasonal rainfall where figure (**a**) shows annual, (**b**) winter, (**c**) summer, (**d**) monsoon, and (**e**) post monsoon rainfall pattern.

We used the coefficient of variation techniques to explore the rainfall variation for all meteorological sub-divisions. The Fig. 2a–e showed the spatial mapping of variations of average annual and seasonal rainfall over India using ordinary kriging interpolation method which is geo-statistical approach. The findings of spatial mapping of rainfall variation showed that the meteorological sub-divisions of Western India were recorded highest rainfall fluctuations. The minimum rainfall fluctuation was registered in Assam and Meghalaya (11.35%) metrological divisions, followed by Sub-Himalayan meteorological divisions like West Bengal (12.25%), Orissa (12.92%) and extreme South Indian divisions like Kerala (14.16%), Coastal Karnataka (14.42%) and North Interior Karnataka (14.67%). This result indicates that these states have been experiencing very less inconsistent rainfall trend for 115 years. While, the highest rainfall variation was found in Saurashtra and Kutch (41.14%), followed by Western Rajasthan (37.75%), Arunachal Pradesh (33.23%) and Gujarat region (30.46%) indicating the irregular occurrences of rainfall throughout the year.

The analysis of distribution and fluctuation of rainfall over Indian meteorological divisions’ show that the occurrence of winter season rainfall was comparatively less than the other seasons during 1901–2015. The maximum variation of rainfall was reported in Konkan & Goa (253.37%), followed by Coastal Karnataka (187.70%), Gujarat region (185.12%), Saurashtra & Kutch (174.88%) and Madhya Maharashtra (162.06%) indicating the unstable incidence of rainfall in these meteorological units. More consistent rainfall with low variations was observed in Himachal Pradesh (41.89%), followed by Arunachal Pradesh (43.15%), Uttarakhand (48.69%) and Assam & Meghalaya (52.21%) meteorological units (Fig. 2c). Result show that, the rainfall in summer season had not recorded a significant variation, whilst the maximum variations of rainfall was observed in the meteorological divisions of Western India. The lowest rainfall variation was found in the meteorological divisions of Northeastern India and extreme South India.

The study of diverge distribution of rainfall over India for monsoon season indicates that the maximum rainfall was registered in Saurashtra & Kutch (42.67% and 157.64%), followed by West Rajasthan (39.93% and 145.35%), Arunachal Pradesh. Whereas, the highest rain fall variation in post-monsoon season was found in Punjab (36.18% and 138.65%), followed by Gujarat region (31.38% and 127.66%) indicating unstable rainfall incidences (Fig. 2d,e). The lowest rainfall fluctuation of monsoon season was recorded in the meteorological divisions of Assam & Meghalaya (12.69%), followed by Orissa (13.34%), Sub Himalayan West Bengal (13.94%) and Coastal Karnataka (15.29%). While, in the post-monsoon season, the lowest rainfall fluctuation was observed in Kerala (26.21%), followed by Tamil Nadu (29.46%) and South Interior Karnataka (36.44%).

### Meteorological sub-division wise trends of Annual and Seasonal Rainfall

We calculated the annual and seasonal rainfall trend for thirty-four meteorological sub-divisions using non-parametric Mann-Kendall test (Table 1). The Table 1 shows the five shades of brown color based on the intensity of z value of Mann-Kendall test at 0.05 significance level which indicates that darker the shade of brown color, higher the negative z value and vice-versa. Results show that five sub divisions for annual rainfall were found in very dark shade zones (Nagaland Mizoram Manipur & Tripura, East Madhya Pradesh, Jharkhand, East Uttar Pradesh, Chhattisgarh and Kerala) which suggests that these sub-divisions were experienced highly negative trend of rainfall (more than −2 of z value). The z value of MK test ranges from 0 to −2 which could be found in eight meteorological sub-divisions, i.e. Bihar, Orissa, Assam & Meghalaya, West Uttar Pradesh, Uttarakhand, Himachal Pradesh, Vidarbha and East Rajasthan (dark brown color in Table 1). The z value having the positive trend varies from 0 to 2 which were observed in the eleven meteorological sub-divisions that implies the increasing nature of rainfall over these divisions, while the six meteorological sub-divisions recorded highly positive trend of more than 2 of z value (very light shade of brown color in Table 1), which indicates the significant increase of rainfall over time in these meteorological units.

**Table 1:** Results of the MK test (Z) and the percentage change estimation for annual and seasonal rainfall for the period of 1901–2015.

Meteorological Sub-divisions	Annual	Monsoon	Post Monsoon	Summer	Winter
Arunachal Pradesh	−1.12	−1.21	−0.54	−0.98	−0.21
Assam & Meghalaya	−1.99	−3.45	0.44	−0.51	−1.33
Nagaland, Manipur, Mizoram & Tripura	−2.74	−1.33	0	3.27	3.01
Sub Himalayan West Bengal & Sikkim	0.32	2.53	−0.08	0.82	−0.06
Gangetic West Bengal	2.41	−1.36	−0.44	−0.28	−0.68
Orissa	−1.19	−2.08	0.37	−0.52	−1.28
Jharkhand	−2.42	−1.6	0.54	2.23	−0.7
Bihar	−1.77	−2.03	−1.24	−0.14	−1.28
East Uttar Pradesh	−2.74	−0.79	−0.98	−0.09	−1.33
West Uttar Pradesh	−1.28	−1.87	−1.42	0.8	−0.79
Uttarakhand	−1.38	0.63	−1.42	0.8	−0.79
Haryana, Delhi & Chandigarh	0.58	0.65	1.1	0	−0.55
Punjab	0.36	−2.38	0.39	0.24	−0.54
Himachal Pradesh	−0.99	0.05	−0.06	2.28	1.16
J & k	0.56	−0.88	−1.62	−0.57	−1.59
West Rajasthan	0.69	0.28	1.25	−0.29	0.55
East Rajasthan	−1.03	−2.23	−0.63	−0.96	−0.53
West Madhya Pradesh	0.02	0.38	−0.16	−0.27	−0.24
East Madhya Pradesh	−2.70	−0.74	−0.24	−0.82	−0.43
Gujarat Region	0.58	0.69	1.5	−0.51	−0.14
Saurashtra & Kutch	1.65	1.52	2.25	−1.34	0.2
Konkan & Goa	2.26	2.26	−1.52	−0.59	−1.79
Madhya Maharashtra	3.32	3.68	−1.82	−2.46	−0.34
Marathwada	0.26	0.24	0.04	−1.49	−1.84
Vidarbha	−0.80	−0.44	0.55	−0.65	−0.79
Chhattisgarh	−3.14	−2.09	0.6	−1.65	−0.39
Coastal Andhra Pradesh	1.08	1.52	−0.12	0.62	−0.5
Telengana	2.01	1.74	−0.38	1.58	2.42
Rayalseema	1.45	1.22	−0.89	2.36	0.57
Tamilnadu	0.65	1.22	0.3	0	0
Coastal Karnataka	2.69	2.31	0.44	−0.64	0.56
North Interior Karnataka	1.45	1.17	−0.82	−2.14	0.86
South Interior Karnataka	2.80	3.93	−0.5	−1.64	0.03
Kerala	−2.15	−2.33	1.73	0.76	0.44
Index of detected trend					
	>2	2-0	0 – (−2)	< −2	

The two meteorological division (Madhya Maharashtra and North Interior Karnataka) for summer season and seven meteorological divisions (Assam & Meghalaya, Orissa, Bihar, Punjab, East Rajasthan, Chhattisgarh and Kerala) for monsoon season were recorded highly negative trend in rainfall having >2 of z value (very dark shade rows in Table 1). On the other hand, no meteorological divisions for winter and post-monsoon season were recorded as highly negative trend (more than >2 of z value) in rainfall, while most of the sub-divisions were detected as negative trend having the z value 0 f 0 to −2 (dark shade rows in Table 1).

### The Change Point Detection analysis for annual and seasonal rainfall

The above-mentioned analysis (Table 1) explained that few meteorological sub-divisions were detected as significant negative trend having the z value of >−2. However, several researchers claimed that the actual trend could not be detected if we apply the MK test on overall hydro-climatic datasets. Therefore, they highly recommended to apply the change detection techniques before the application of MK test. Hence, we utilized change detection methods like Pettitt test, SNHT test and Buishand range test for detecting the abrupt change point in the rainfall datasets in thirty-four meteorological sub-divisions (Supplementary Table 1). The Supplementary Table 1 shows that the annual and seasonal rainfall of all meteorological sub-divisions had the abrupt change point which were detected by mentioned three change detection techniques. Furthermore, we selected the change point for annual and seasonal rainfall in each sub-divisions based on the performances (p value) of these tests (Supplementary Table 1). Therefore, these abrupt change points suggest that the rainfall datasets had no monotonous trend. The selected change point for seven meteorological sub-divisions (East Uttar Pradesh, West Uttar Pradesh, Punjab, and Gujarat region, Saurastra & Kutch, Coastal Andhra Pradesh and Tamil Nadu) were after 1990s. The nineteen meteorological sub-divisions had the abrupt change point during the period of 1950–1980. The abrupt change point year for the rest of the meteorological divisions were detected before 1940.

### Change point wise annual and seasonal variation analysis

**Figure 3:**
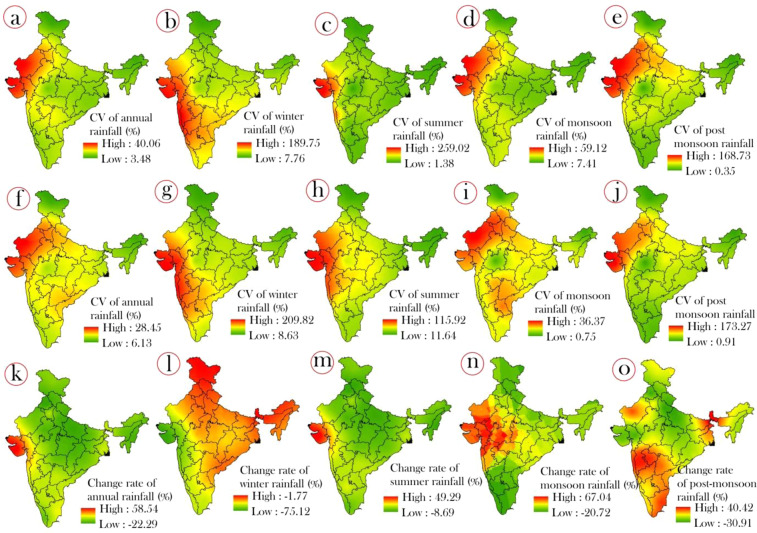
Spatial variation of rainfall measured using the coefficient of variation for pre-change point where figure (**a**) shows annual rainfall, (**b**) winter, (**c**) summer, (**d**) monsoon, and (**e**) post monsoon; post-change point where figure (**f**) shows annual rainfall, (**g**) winter (**h**) summer (**i**) monsoon and (**j**) post monsoon; spatial changes in the rate of rainfall where figure (**k**) shows annual rainfall, (**l**) winter (**m**) summer (**n**) monsoon and (**o**) post monsoon.

We computed the annual and seasonal rainfall variation in pre and post change point wise for all meteorological sub-divisions to explore the dynamics of the intensity of annual and seasonal rainfall variations after the change point. Therefore, we can consider this change point wise rainfall variation analysis as a validating method for the relevancy of uses of the change point methods and further research. Hence, to prove this statement, we computed and prepare the spatial map of annual and seasonal rainfall variation for pre change point (Fig. 3a–e) and post change point (Fig. 3f–j) using coefficient of variation method. Results show that highest fluctuation (25% to 239.02%) in annual and seasonal rainfall was observed in the sub-divisions of Western India and North-Western India (3a-e). Whereas, the minimum fluctuation in annual and seasonal rainfall having the CV of 0.36% to 7.76% was observed in the sub-divisions of North Eastern India, Eastern India and Northern India indicating the consistent rainfall occurrence in these region.

The findings of annual and seasonal rainfall analysis in post change point phase show that the large areas of the country like the sub-divisions of Western India, Central India, and South Western India observed high fluctuation having the CV of 22% to 209.82%. In case of monsoon and post monsoon season, the sub-divisions of North Western were experienced by high variation of rainfall suggesting the high tendency of inconsistently rainfall occurrences in these regions. While the lowest rainfall variation having the CV of 0.75% to 11.64% was recorded in sub-divisions of North-Eastern India, North India, East India of winter and summer rainfall and Central India of Post monsoon rainfall. The intensity of minimum rainfall variation range was increased in post change point phase which suggests that inconsistency rainfall events were observed. However, in the post change point phase, the area coverage of lower variation of rainfall incidences were reduced significantly that implies the climate change and validation of the application of change point detection methods.

### Rainfall change rate analysis

In the present study, we computed the rainfall change rate for annual and seasonal rainfall in all meteorological sub-divisions based on the calculation between the rainfall data of pre and post change point phase. The Fig. 3k–o shows the spatial mapping of rainfall change rate for annual and seasonal rainfall. Results show that sub-divisions of Western India were observed highest change rate (47% to 58.54%) in annual rainfall, while the sub-divisions of whole country except West India were registered the highest change rate (−0.5% to −1.77%) in winter rainfall. In case of summer and monsoonal rainfall, the sub-divisions of western were observed highest negative change rate (34.22% to 67.04), while the South India and North East India were recorded highest negative change rate in post monsoon rainfall. Furthermore, the North India and Central India were recorded minimum change rate, whereas, the Western India of winter and post-monsoon rainfall. This analysis signifies that the amount of rainfall occurrences was decreased significantly after change point.

### Change point wise seasonal rainfall trend analysis

We applied MK test on the datasets of pre and post change point of seasonal rainfall in each of the meteorological sub-divisions as per the recommendation of many scientists. The Table 2 reported the seasonal rainfall trend for pre change point in all sub-divisions Results show that eight meteorological sub-divisions in monsoon, six sub-divisions in post monsoon, fourteen sub-divisions in both summer and winter season were recorded negative trend having the z value of 0 to −2 (dark shade of brown color rows in Table 2). While, three sub-divisions in summer and four sub-divisions in winter seasons were recorded highly negative trend having z value of > −2 (very dark shade of brown color rows in Table 2). Rests of the meteorological sub-divisions were detected as positive trend (lighter shade of brown color in Table 2) except one division (Sub Himalayan West Bengal & Sikkim) which detected has no trend. From the analysis, it can be stated that non-monsoonal seasons were recorded as declining trend.

**Table 2:** Meteorological subdivision wise trend for pre-change point.

Meteorological sub-divisions	Z value for Pre Change Point			
	Monsoon	Post Monsoon	Summer	Winter
Arunachal Pradesh	−0.41	−1.31	−0.15	−0.26
Assam & Meghalaya	0.11	0.52	−0.36	−1.52
Manipur, Mizoram, Nagaland & Tripura	2.07	0.59	0.30	0.40
Sikkim &Sub Himalayan West Bengal	0.00	0.00	0.00	0.00
Gangetic West Bengal	−1.07	0.73	−2.55	−0.56
Orissa	1.03	1.44	−1.79	−0.45
Jharkhand	0.49	1.39	−1.61	0.00
Bihar	0.08	1.11	−0.31	1.72
East Uttar Pradesh	1.07	1.40	0.02	−0.54
West Uttar Pradesh	1.40	1.24	0.35	−1.14
Uttarakhand	0.69	0.98	0.97	0.83
Haryana, Delhi & Chandigarh	−0.21	−0.14	−0.69	−0.59
Punjab	2.31	0.02	0.06	−0.50
Himachal Pradesh	1.19	2.34	1.74	2.04
J & k	−0.56	0.13	−0.41	0.18
West Rajasthan	0.57	−0.75	−1.04	0.41
East Rajasthan	1.64	−0.07	−1.19	−1.86
West Madhya Pradesh	2.66	0.22	−1.12	−1.46
East Madhya Pradesh	1.18	0.66	−0.50	1.29
Gujarat Region	−0.25	1.17	−2.60	−2.45
Saurashtra & Kutch	0.30	1.49	−2.74	−2.90
Konkan & Goa	0.46	0.68	−0.73	0.22
Madhya Maharashtra	0.62	0.24	−0.26	0.02
Marathwada	−1.04	0.30	−0.23	0.84
Vidarbha	1.76	1.13	0.90	−1.08
Chhattisgarh	2.18	1.32	0.08	−0.91
Coastal Andhra Pradesh	1.36	−0.21	−0.29	0.30
Telengana	0.72	1.24	1.11	0.41
Rayalseema	0.33	−0.62	0.66	−3.69
Tamilnadu	0.45	−0.66	−1.97	−2.94
Coastal Karnataka	−0.03	0.66	1.20	−1.06
North Interior Karnataka	−1.24	0.15	0.05	0.81
South Interior Karnataka	−0.59	−0.15	−0.11	−0.61
Kerala	−0.66	−1.23	3.12	−0.12
Index of detected trend				
	>2	2-0	0 – (−2)	< −2

The Table 3 showed the trend analysis using MK test for post-change point seasonal rainfall. Seventeen meteorological sub-divisions in monsoon, sixteen sub-divisions in post monsoon, seventeen sub-divisions in summer and twenty one sub-divisions in winter seasons were observed the negative trends having the z value of 0 to −2 indicating that huge amount area was recorded declining rainfall than the pre-change point phase (dark shade of brown color rows in Table 3). The increasing of meteorological divisions which have the negative trend from the pre change point (Table 3). This circumstance implies that the declining trend of rainfall was increased manifold after post change point which shows the sign of climate change. On the other hand, rest of the meteorological sub-divisions were experienced the insignificant positive trend having the z value of <0.5.

**Table 3:** Meteorological subdivision wise trend for post-change point.

Meteorological sub-divisions	Z Value for Post Change Point			
	Monsoon	Post Monsoon	Summer	Winter
Arunachal Pradesh	0.28	−0.72	−0.31	−0.64
Assam & Meghalaya	0.67	0.36	−0.36	−0.74
Manipur, Mizoram, Nagaland & Tripura	0.19	0.44	−0.51	−1.33
Sikkim &Sub Himalayan West Bengal	−1.87	0.00	3.27	3.01
Gangetic West Bengal	−0.77	−0.08	0.82	−0.06
Orissa	1.08	−0.44	−0.28	−0.68
Jharkhand	0.30	0.37	−0.52	−1.28
Bihar	−0.13	0.54	2.23	−0.70
East Uttar Pradesh	−1.43	−1.24	−0.14	−1.29
West Uttar Pradesh	−0.63	−0.98	−0.09	−1.33
Uttarakhand	1.19	−1.42	0.80	−0.79
Haryana, Delhi & Chandigarh	−1.69	−1.42	0.80	−0.79
Punjab	1.04	1.10	0.00	−0.55
Himachal Pradesh	0.70	0.39	0.24	−0.54
J & k	0.14	−0.16	0.13	0.26
West Rajasthan	−0.34	−0.06	2.28	1.16
East Rajasthan	0.26	−1.62	−0.57	−1.59
West Madhya Pradesh	−0.44	1.25	−0.29	0.55
East Madhya Pradesh	−1.25	−0.63	−0.96	−0.53
Gujarat Region	−0.87	1.50	−0.51	−0.14
Saurashtra & Kutch	0.00	2.25	−1.34	0.20
Konkan & Goa	−0.89	−1.52	−0.59	−1.79
Madhya Maharashtra	0.71	−1.82	−2.46	−0.34
Marathwada	−1.12	0.04	−1.49	−1.84
Vidarbha	0.79	0.55	−0.65	−0.79
Chhattisgarh	−0.28	0.60	−1.65	−0.39
Coastal Andhra Pradesh	0.94	−0.12	0.62	−0.50
Telengana	−1.44	−0.38	1.58	2.42
Rayalseema	0.18	−0.89	2.36	0.57
Tamilnadu	−0.64	0.30	0.00	0.00
Coastal Karnataka	−0.09	0.44	−0.64	0.56
North Interior Karnataka	−0.58	−0.82	−2.14	0.86
South Interior Karnataka	0.26	−0.50	−1.64	0.03
Kerala	0.28	1.73	0.76	0.44
Index of detected trend				
	>2	2-0	0 – (−2)	< −2

#### Innovative trend analysis for seasonal rainfall

**Figure 4:**
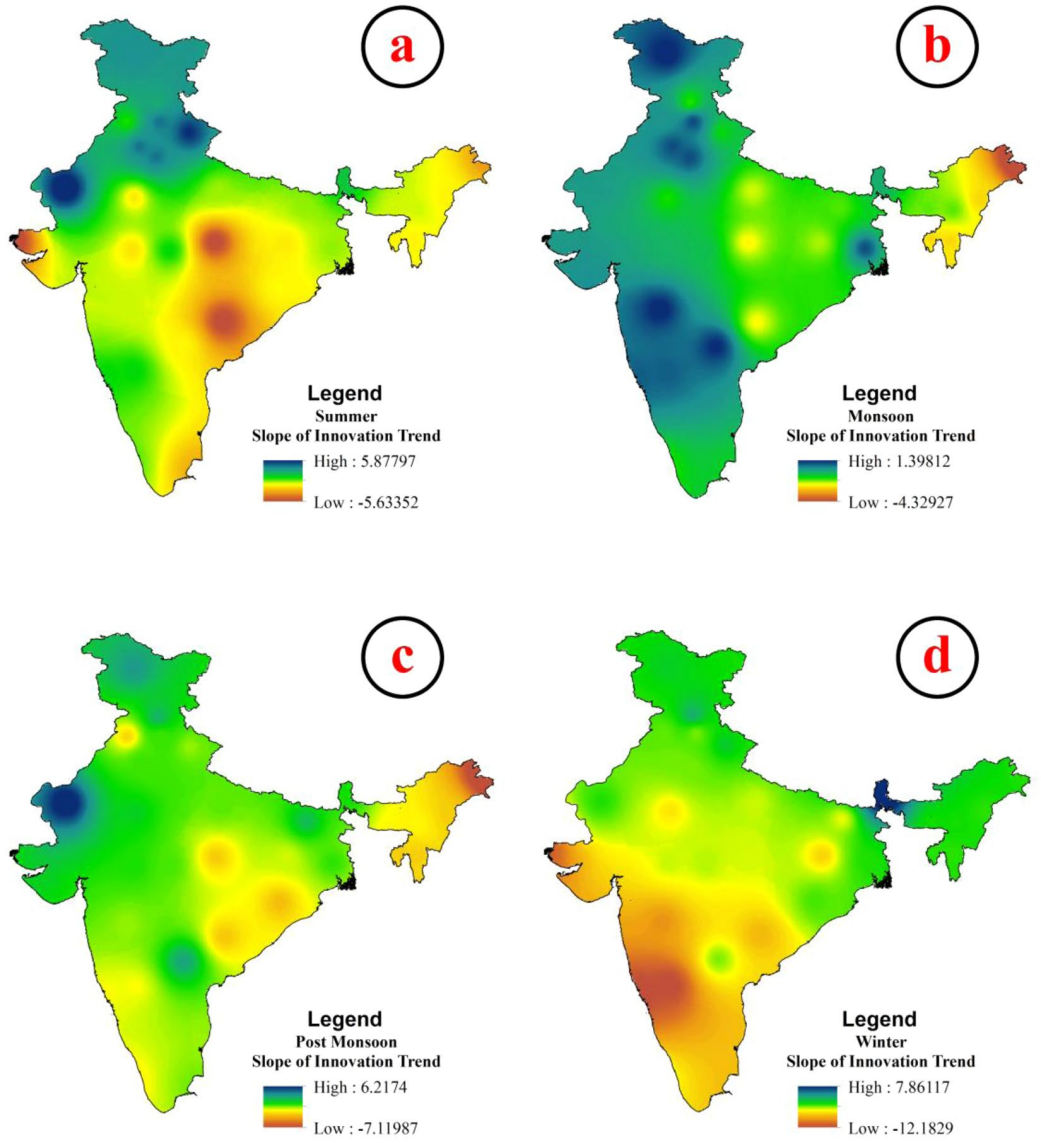
The spatial variation of slope of innovative trend for (**a**) summer, (**b**) monsoon, (**c**) post monsoon and (**d**) winter.

Several researchers suggested that non-parametric statistical techniques like mann-kendall test has many drawbacks like the presence of serial correlation within the data sets, non-linearity and most importantly sample size which could have ability to influence the result. Therefore, Sen^55^ developed innovative trend method which can overcome the mentioned drawbacks, especially the problem sample size. Sen^55^ reported that innovative trend can effectively able to detect the trend on any numbers of sample size and presence of serial correlation. Hence, we used innovative trend to calculate the trend for seasonal rainfall (winter, summer, monsoon and post monsoon) in thirty-four meteorological sub-divisions (Supplementary Figure 1–4). We computed D value of innovative trend to compare the intensity of trend achieved by MK test. The spatial mapping using D values of innovative trend for seasonal rainfall was presented in Fig. 4a–d. However, the Supplementary Table 2 showed the slope value of innovative trend for seasonal rainfall in all meteorological sub-divisions. The findings indicate that the negative trend was detected in the sub-divisions of North Eastern, Central and Southern India for summer season. The results were quite identical with the findings of MK test, but the magnitudes of the trend were different which stated that the region was experienced strong negative trend (Fig. 4). Although the highly positive trend was detected in the sub-divisions of Rajasthan part and Jammu-Kashmir region which does not imply that the rainfall occurrences was increased, but the regions were received more or less consistent amount of rainfall throughout the time periods, while little amount of rainfall was increased in few years over these regions which is the reason for positive trend. However, in case of monsoon rainfall, the negative slope of innovative trend was detected in the sub-divisions of North Eastern part, Eastern part and some parts of the central India, whereas, rest of the sub-divisions were detected as insignificant positive slope of trend. The sub-divisions of North Eastern states, Bihar, Orissa, Jharkhand, Western Ghat and Punjab regions were experienced very strong negative slope of innovative trend, on the other hand, rest of the part were recorded insignificant positive slope of trend for post monsoon rainfall. In case of winter rainfall, the meteorological sub-divisions of Central part of India, Southern India, Western Ghat regions were observed the negative trend, while the sub-divisions of North Eastern, Western and Eastern part of India were experienced positive slope of trend. Therefore, the findings of MK test and innovative trend analysis were highly identical, however, few states where no significant trend were detected using MK test, but in the case of innovative trend, those region were come under negative trend. However, the findings from both trend detected methods clearly stated that India has been experiencing fewer downpours than the expected rainfall since last 30 years that clearly points out about the climate change. Several researches established that innovative trend can able to detect trend effectively over the others non-parametric test^18,99,100^^,^. Therefore, we considered innovative trend as a tool of intensity of trend measurement over the other techniques and results show that the highly negative trend was detected in most of the sub-divisions indicate about the climate change.

### Micro level rainfall change rate analysis

**Figure 5:**
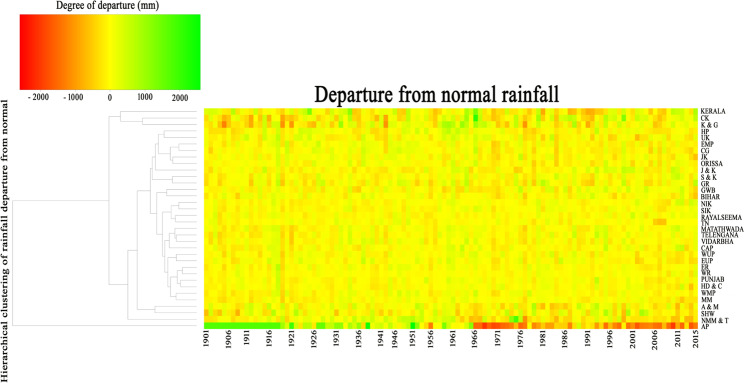
Heatmap represents the departure of rainfall from normal rainfall for all meteorological subdivision. (N.B. CK- Coastal Karnataka, K & G – Konkan and Goa, HP- Himachal Pradesh, UK- Uttarakhand, EMP- Eastern Madhya Pradesh, CG- Chhattisgarh, JK – Jharkhand, J & K – Jammu and Kashmir, S & K- Saurashtra and Kachcha, GR – Gujarat region, GWB – Gangetic West Bengal, NIK – North Interior Karnataka, SIK – South Interior Karnataka, TN – Tamilnadu, CAP – Coastal Andhra Pradesh, WUP – western Uttarpradesh, EUP – Eastern Uttarpradesh, ER – Eastern Rajasthan, WR – Western Rajasthan, HD & C- Haryana Delhi and Chandigarh, WMP – WesternMadhyapradesh, MM – MadhyaMadhyapradesh, A & M- Assam and Meghalaya, SHW – Sub Himalaya West Bengal, NMM & T- Nagaland, Manipur, Mizoram and Tripura, AP- Arunachal Pradesh.

In the present study, we attempted to analyze the change rate of annual rainfall for each and every year in thirty-four meteorological sub-divisions. We computed the change rate by calculating the departure of year wise average rainfall from the long-term average rainfall. The heat map was used to show the dynamics of year wise rainfall change rate for all sub-divisions (Fig. 5). This heat map was generated from R software (version 3.5.3) (https://cran.r-project.org/bin/windows/base/old/3.5.3/) using the *ggplot2* package^101^ (https://cran.r-project.org/web/packages/ggplot2/index.html). The intensity of change rate was represented by the shades of red and green color indicating the highly negative change rate and vice versa. Results show that the after 1970, all meteorological sub-divisions were observed the negative departure in almost all years from long-term average rainfall by 50 mm.−2000 mm. (Fig. 5). While the sub-divisions of North-Eastern India and North India were recorded positive change rate only for few years because of the occurrences of excessive rainfall that was happened occasionally by monsoon burst, ELSO effect (Kripalini *et al*. 2003) and local climatic effect. However, the highest negative change rate was observed in the sub-divisions like Arunachal Pradesh, Nagaland Manipur, Mizoram & Tripura, Kerala, Western Uttar Pradesh, Rajasthan, Uttarakhand and Himachal Pradesh by more than −2000 mm. While the positive change rate was mainly detected before change detection year or 1970 in all sub-divisions. The positive change rate was varied from 0–2000 mm. However, few sub-divisions like Kerala, Nagaland Manipur Mizoram & Tripura, and Coastal Karnataka.

### Rainfall prediction and forecasting

**Figure 6:**
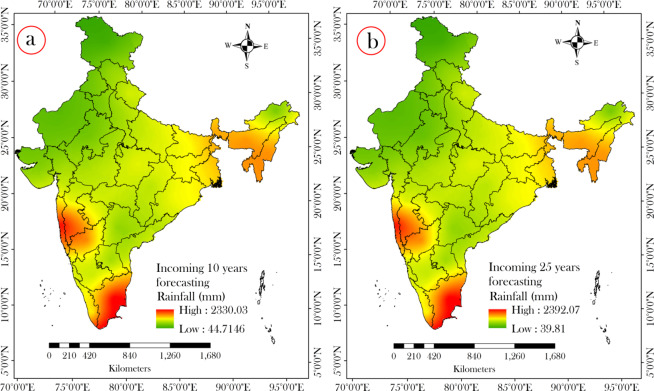
Spatial rainfall forecast for 2020 (**a**) and 2030 (**b**).

The above mentioned analyses show that most of the meteorological sub-divisions of India were experienced significant decrease in rainfall and this decrease was become stronger after the change point. Therefore, the situation of rainfall incidence became critical post change point. Therefore, if the present situation of rainfall occurrences continues with the same intensity, the intensity of rainfall occurrences and their distribution in future will be more worsen. Therefore, the prediction and forecasting of rainfall is become essential for water resource management and planning. Several soft computing machine learning techniques are available for predicting and forecasting rainfall, but MLP based artificial neural network has become widely popular among the researchers as it is easy to compute and generate high quality product^102,103^. In this study, we applied MLP-ANN on the annual rainfall data for prediction. We set the parameters of artificial neural network models again and again until the best prediction had not achieved which we evaluated using RMSE techniques. We fixed the model’s parameters and applied on the rainfall of thirty-four meteorological sub-divisions for prediction. In some cases, we changed some of models parameters for predicting in rainfall. However, the Supplementary Table 3 shows the plot between the best MLP-ANN generated model and observed rainfall for thirty four meteorological sub-divisions that indicates that there is close adjacency between predicted and observed rainfall. The error measures like RMSE and MAE (in mm.) were used to quantify the closeness between predicted and observed rainfall. The Supplementary Table 4 reports the 15 years rainfall forecasting up to 2030 for all meteorological sub-divisions. We selected the rainfall of 2020 and 2030 as the representative for spatial mapping (Fig. 6) (for the rainfall forecasting data of all years was presented in Supplementary Table 4). The findings of spatial mapping of future forecasting show the more declining amount of rainfall.

In 2020, the maximum rainfall incidence will be observed in the sub-divisions of Coastal Karnataka, Konkan & Goa, Kerala (>2500 mm rainfall) and the minimum rainfall event will be placed on the West Rajasthan, Tamil Nadu, East Rajasthan, Rayalseema (<400 mm rainfall) (Fig. 6a). On the other hand, in 2030, the highest rainfall will be occurred in the sub-divisions of Coastal Karnataka, Konkan & Goa, Kerala (>2000 mm rainfall), while the lowest rainfall event will be found in the sub-divisions like West Rajasthan, East Rajasthan, Tamilnadu, Rayalseema (<300 mm rainfall) (Fig. 6b). The findings of future forecasting analysis point out that the occurrences of rainfall will be decreasing gradually for some meteorological stations, while the rainfall will be increased in some meteorological sub-division. The places of rainfall occurrences over India will be remained as like identical pattern of present and earlier rainfall incidences. The findings also show that the extreme rainfall events will be increased in future that will be lead to flooding situation.

### Causes of rainfall variation

**Figure 7:**
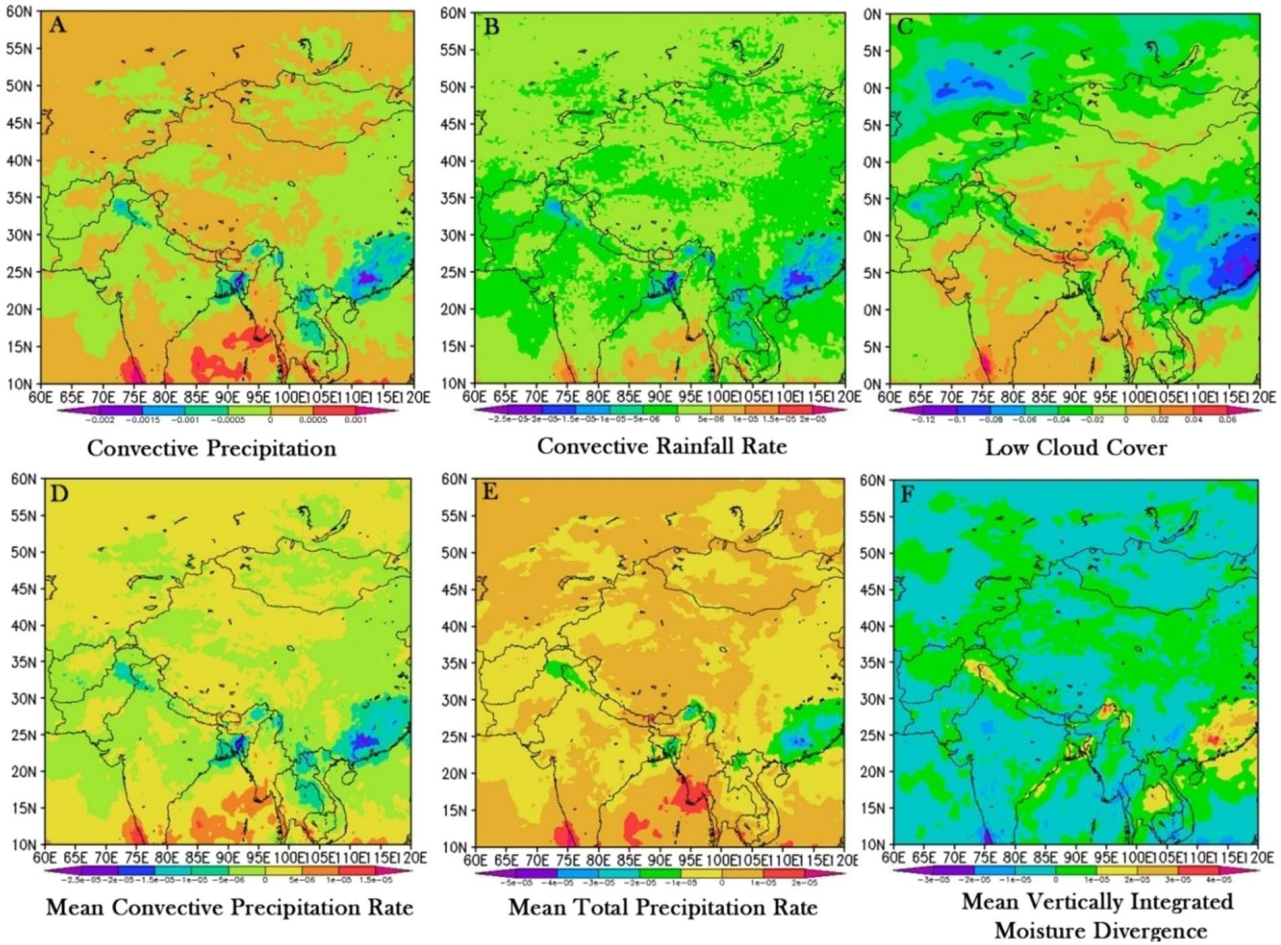
Spatial variations of differences in (**a**) convective precipitation in monsoon season, (**b**) convective rainfall rate in winter season, (**c**) low cloud cover, (**d**) mean convective precipitation rate, (**e**) mean total precipitation rate, and (**f**) mean vertically integrated moisture divergence between the recent period of 2001–2015 and 1979–2000.

India encompasses large areas with moderate to high convective precipitation, while low convective rainfall rate occurred which was brought excessive moisture from the Indian Ocean to the in land areas, which is unfavorable for the formation of rainfall (Fig. 7a–f). This caused a decreased in mean rainfall during monsoon season, which has been distributed in the extreme southern and mid-central divisions of the country (Fig. 7). The northeasterly wind was strengthened in the whole country, which reduced the invasions of cooler air, led to decline in rainfall all over India in the winter season. By contrast, most of the divisions had moderate to high mean convective precipitation rate, which increased rainfall to some extent. The low cloud has increased all over the India, and a few cloud covers will enhance the consolidation impact of atmosphere on the solar radiation, and hence it leads to declining rainfall trend. Moreover, high mean total precipitation rate has been influencing across the country except for eastern division, which triggered uneven downdraft pattern and leading to more clear sky days during the recent study period (1979–2017). Most of the regions in India exhibited a declining vertically integrated moisture divergence which triggered by a significant decreasing rainfall changes.

## Discussion

Marumbwa *et al*.^104^ suggested that the analysis of historical rainfall trend is very crucial in several fields like water resource management, sustainable agricultural planning, ecosystem management and health sector. The rainfall data of 115 years were used to investigate the variability of annual and seasonal rainfall in very detail and analyzed the trend in several ways for thirty-four meteorological sub-divisions. The findings of 115 years annual and seasonal rainfall variation analysis show that the highest rainfall variation was found in the sub-divisions of Western India of annual and summer, Western India and South Western India of winter, North India and Western India of monsoon and Western, North Western and some parts of Eastern India of post monsoon rainfall. While the lowest variation was found in the sub-divisions of North Eastern and Eastern India. The identical result was reported by several previous studies^32,45,66,71,105^. The findings of MK test on annual and seasonal rainfall report that thirteen meteorological sub-divisions were observed negative trend, while rest of the sub-divisions were recorded positive trend. Rajeevan *et al*.^106^ and Guhathakurta and Rajeevan^46^ reported that Jharkhand, Chhattisgarh and Kerala were observed negative trend, while eight sub-divisions like Gangetic West Bengal, Coastal Andhra Pradesh, Kanakan & Goa, North Interior Karnataka, Rayalessema, Jammu &Kashmir were recorded decreasing trend over time. These results are totally identical with the findings of the MK test of the present study (Very dark shade of brown color rows in Table 1). Mirza *et al*.^107^ reported that the annual and seasonal rainfall was more or less consistent in sub-divisions sub-himalayan Bengal and Gangetic Bengal which was by and large similar with the findings of MK test in the present study (Table 1).

Mondal *et al*.^32^ used Pettitt test and SNHT test for detecting change point in the annual rainfall for all meteorological sub-divisions for their study and reported that twenty-one sub-divisions had the change point using Pettitt test between 1950–1966. While we detected eighteen sub-divisions that have change point between 1950–1966 (Supplementary Table 1). Goyal^98^ documented that the most probable change point was 1959, while in the present study, most of the change points were detected in between 1950–1966 which can conclude that the findings of present work are identical with the work of Goyal. Therefore this study concludes that 115 years annual rainfall had not the monotonous trend and it needs for further research to detect the trend and its magnitude accurately. Based on which planners and scientists can propose the developmental and management plan.

Furthermore, MK test was employed on the annual and seasonal rainfall data of both pre and post change point phase to detect the exact and accurate trend as several scientists recommended to apply MK test based on the analysis of change point to achieve the accurate trend^34,38,108^. The misleading results could be found if the MK test applies first^38^. However, The findings of MK test on the annual and seasonal rainfall for pre change point phase stated that among the 34 meteorological divisions, 8 sub-divisions of each monsoon and post monsoon, 17 sub-divisions of summer and 18 sub-divisions of winter season were detected insignificant negative but trend (Table 2). Parthasarathy and Dhar^109^ documented that most of the sub-divisions were recorded positive trend for the period 1901–1970 that was the change point year for the most of the sub-divisions in the present study (Table 2 and Supplementary Table 1).

The findings of the MK test for post change point phase showed that the negative significant trend was detected in the 17 meteorological sub-divisions of monsoon, 15 sub-divisions of post monsoon, 19 sub-divisions of summer and 21 sub-divisions of winter season (Table 3). Jain and Kumar^110^ reported that 4 river basins of India were detected as increasing trend, while 13 river basins were observed significant negative trend in monsoon rainfall. In case of annual rainfall, 15 river basins were recorded negative trend. Therefore, we can state that these findings are supporting the present work. Guhathakurta *et al*.^111^ reported that significant negative trend was found in NW and Central and Peninsular India for the period of 1951–2011. Several studies also reported that the significant amount of rainfall was decreased over time, especially after 1950^44,46,81,105,112^.

To the best of authors’ knowledge, the application of recently developed innovative trend analysis to detect rainfall trend was very rare. We found the work of Machiwal *et al*.^113^ who applied innovative trend analysis to detect trend in rainfall and temperature of Indian arid region. Machiwal *et al*.^113^ documented that increasing trend for monsoon rainfall was found in the arid region and this result is identical with the findings of the present study. However, the findings of Innovative trend analysis on annual and seasonal rainfall showed that negative trend was observed in the sub-divisions of NE, E, SE and S part of summer, NE and some parts of E India of monsoon, NE, E India of post-monsoon and W, SW, S India of winter rainfall. The identical finding was found in the work of Mondal *et al*.^32^. Taxak *et al*.^96^ studied grid wise rainfall trend over India and documented that most of grids were received negative trend except seven grids which were observed positive trend. The findings of this study are totally identical with the present work.

Dash *et al*.^53^ and Kumar *et al*.^11^ documented that frequency of intense rainfall in many parts of Asia has increased, while the amount of rainfall and number of rainy days has decreased significantly. For this reason, the year wise departure study revealed that some of the years were observed positive departure from the long-term average rainfall by more than 1000 mm rainfall (Fig. 5). Even the identical findings can be found in the work of Goswami *et al*.^40^ and they reported that the frequency and magnitude of extreme rainfall has amplified significantly, while the frequency and intensity of moderate rainfall has observed noteworthy decreasing trend.

In the present study, the change rate was maximum in the meteorological sub-divisions of Western and Central India in annual and monsoon rainfall, whereas the maximum change rate (%) for winter season was in the sub-divisions of Central, North East and Eastern India. Mondal *et al*.^32^ reported that many parts of Western India and Central India were received very high negative change rate for monsoon rainfall, while Central and North Eastern parts of India were observed highly decreasing change rate. Furthermore, it can be concluding from the analysis that North Eastern part of India has been observed the worst effect of climate change than any other parts of India.

In the present study, the annual rainfall for thirty-four meteorological sub-divisions were predicted and forecasted up to 2030. In addition, the rainfall forecasting for 2030 showed an expected decline of about 5–10% in the overall rainfall of India (Fig. 6). However, numbers of studies were conducted to predict and forecast rainfall for India based on the average rainfall and they did not consider studying for all parts of the India. Guhathakurta^114^ predicted the rainfall using neural network for Kerala. While Chakraborty *et al*.^49^ predicted the south-west monsoon for India. They used average time series annual rainfall dataset of India. But in the present study, we considered rainfall datasets for thirty-four meteorological sub-divisions. Therefore, the findings of this study would not be generalized; rather it would be more accurate and can be act as the foundation of the developmental planning.

The temporary change in rainfall distribution significantly distresses the agricultural production. It will increase the drought protection and resilience plans under the changing climate conditions. Intergovernmental Panel on Climate change (IPCC) reported that upcoming changes in climate is to be likely to distress agriculture that will amplify the chance of hunger and water paucity, as well as the instructions on quicker thawing of glaciers^115^. Gosain *et al*.^116^ pointed that the amount of freshwater in the river of India is probable to be diminish because of changing climate. This reduction, along with growing population might unfavourably affect a large population in India by 2050.

Large-scale ocean-atmospheric changes derived from the ECMWF ERA5 re-analysis data depicted that compared between 1979–2000 to 2001–2015 period, an increasing/decreasing convective precipitation rate, enhanced low cloud cover and inadequate moisture variance in the Indian ocean being transported to the northwest direction might have highly influenced the rainfall trend in India.Therefore, it is expected that this study will provide an insight for the management and development of agriculture and water resources in India to overcome the possible impacts of the climate change.

## Conclusion

The basic and essential requirement for the management and planning of water resource, sustainable agricultural development and other sectors is the exploration of the spatiotemporal distribution and changing pattern of rainfall in any places. Hence, the present study investigated the variability and trend analysis of annual rainfall in several ways like overall data, change point wise (pre and post change point) using 115 years long-term annual and seasonal rainfall data of thirty-four meteorological sub-divisions. The present study shows that the overall annual and seasonal variability of rainfall was highest in the sub-divisions of Western India, while the lowest variability was found in Eastern and North India. The findings of MK test on overall annual and seasonal rainfall reports that the sub-divisions of North-East, South and Eastern India were detected significant negative trend, while the sub-divisions like Sub-Himalayan Bengal, Gangetic Bengal, Jammu & Kashmir, Konkan & Goa, Madhya Maharastra and Marathwada were recorded positive trend. Furthermore, the change detection techniques were utilized and selected the change point based on the performances of the techniques. The most probable change points were detected in between 1950–1966. Based on the change point year, the rainfall variability and trend analysis were again carried out for pre and post change point phase. The rainfall variability was increased significantly in most of the meteorological sub-divisions after post change point and similar kinds of findings were found when the rainfall trend was analyzed for post change point. To get better results of trend analysis, the innovative trend analysis was employed. The finding shows that most of the sub-divisions were recorded significant negative trend. Even some of the sub-divisions were detected as no trend using MK test, but the trend was detected using innovative trend analysis. However, the micro level change rate analysis was used in the present study and the results show that after 1960, most of the meteorological sub-divisions were recorded more than −500mm rainfall departure from the long-term average rainfall in many years, while few years were detected positive departure in few sub-divisions. Therefore, from the detail analysis, it is established that almost all of the sub-divisions were detected the negative trend and high variability after 1970. Even the year wise study also revealed that in which year and how much rainfall amount was departed. Hence, these detail information regarding historical data for whole country are very beneficial for the planning. One of the most striking features of developmental planning in the recent time is the forecast of the incoming event which can be found in any sector like finance, water resource and most importantly climatology. Therefore, in the present study, the rainfalls for all meteorological sub-divisions were forecasted using the advance AI models like artificial neural network. The findings of the rainfall forecasting show that 15% of rainfall will be declined in 2030 that indicates the alarming situations will be appeared for both environment and living world.

The economy of India is totally dependent on the rainfall either it is agriculture or industry. Therefore, the water resource is essential part of the progressive economy of India. But due to climate change, the world rainfall pattern has been disturbed. Therefore, many studies were carried out in the developed countries to quantify the pattern of the rainfall changes and formulate the management plan accordingly. But in case of India, very fewer studies were done to do so. The present study provides information in all aspects like rainfall variability and trend for overall and change point wise for annual and seasonal rainfall, rainfall change rate since change point year, year wise departure, and future rainfall and most importantly this study analyzes the causes of rainfall changes in India. Technically, the present study used several sophisticated techniques which have been admired worldwide by the scientists for providing high precision results. This type of studies has not been conducted for whole India. Therefore, the present study can be the full package and should be very much helpful to the Indian planners to proposing plans for small and large scale regions.

To formulate the management plan for the sustainable development of water resource based sectors and environment, the scientist of others countries can conduct the research like the present study as they need lots of information for developing plan regarding historical, present and future data which can be in any field like hydrology, climatology.

However, in the present study, we considered thirty-four meteorological sub-divisions for the research, but to be more accurate, micro level data like district wise data should be incorporated. Then the very high precision micro level management plan will be achieved. Even, the grid wise rainfall study using very advanced microwave remote sensing technology will be very useful for the planners. The ensemble machine learning techniques, deep learning techniques like long-short-term memory (LSTM) network can be used to achieve very high quality forecasting data.

## Supplemenatry information

Supplemenatry information.
