# Supplementary material for: Analyzing trend and forecasting of rainfall changes in India using non-parametrical and machine learning approaches
Source: Sci Rep. 2020 Jun 25;10:10342. doi: 10.1038/s41598-020-67228-7 (PMC7316787; doi:10.1038/s41598-020-67228-7)
Supplement: Supplementary file 1 — Supplemenatry information. [file 41598_2020_67228_MOESM1_ESM.docx]

**Analyzing trend and forecasting of rainfall changes in India using non-parametrical and machine learning approaches**

Bushra Praveen^1^, Swapan Talukdar^2^, Shahfahad^3^, Susanta Mahato^2^, Jayanta Mondal^2^, Pritee Sharma^1^, Abu Reza Md. Towfiqul Islam^4^, Atiqur Rahman^3*^

^1^Research Scholar, School of Humanities and Social Sciences, Indian Institute of Technology Indore, Simrol, Indore 453552, India. Email-[bushraparvn@gmail.com](mailto:bushraparvn@gmail.com)

^1^Associate Professor, School of Humanities and Social Sciences, Indian Institute of Technology Indore, Simrol, Indore 453552, India. Email- [psharma@iiti.ac.in](mailto:psharma@iiti.ac.in)

^2^Research Scholar, Department of Geography, University of Gour Banga, Malda, India. Email- [swapantalukdar65@gmail.com](mailto:swapantalukdar65@gmail.com)

^2^Research Scholar, Department of Geography, University of Gour Banga, Malda, India. Email- [mahatosusanta2011@gmail.com](mailto:mahatosusanta2011@gmail.com)

^2^Research Scholar, Department of Geography, University of Gour Banga, Malda, India. Email- [jayantamondal007@gmail.com](mailto:jayantamondal007@gmail.com)

^3^Research Scholar, Department of Geography, Jamia Millia Islamia, India Emails: [fahadshah921@gmail.com](mailto:fahadshah921@gmail.com)

^3^ Professor, Department of Geography, Jamia Millia Islamia, India . Emails: [ateeqgeog@yahoo.co.in](mailto:ateeqgeog@yahoo.co.in)

^4^Associate Professor, Department of Disaster management, [Begum Rokeya University, Rangpur](https://www.researchgate.net/institution/Begum_Rokeya_University_Rangpur) 5400, Bangladesh. Email- towfiq_dm@brur.ac.bd

* Corresponding author

**List of Supplementary Tables**

**Supplementary Table 1** The abrupt change point year for the meteorological divisions using SNHT test, Pettitt test and Buishihand’s range test.

| **Meteorological sub-divisions** | **Changepoint based on SNHT test** | **Changepoint based on the Pettitt test** | **Changepoint based on a Buishand׳s range** | **Selected change point (Year) based on the performance of the test** |
| --- | --- | --- | --- | --- |
| Arunachal Pradesh | 1965 | 1965 | 1968 | 1965 |
| Assam & Meghalaya | 1961 | 1961 | 1961 | 1961 |
| Nagaland, Manipur, Mizoram & Tripura | 1976 | 1961 | 1976 | 1976 |
| Sub Himalayan West Bengal & Sikkim | 1908 | 1910 | 1910 | 1910 |
| Gangetic West Bengal | 1974 | 1967 | 1967 | 1972 |
| Orissa | 1968 | 1966 | 1966 | 1966 |
| Jharkhand | 2013 | 1966 | 1966 | 1966 |
| Bihar | 2013 | 1954 | 1954 | 1954 |
| East Uttar Pradesh | 1995 | 1990 | 1990 | 1990 |
| West Uttar Pradesh | 1974 | 1990 | 1990 | 1990 |
| Uttarakhand | 1976 | 1976 | 1976 | 1976 |
| Haryana, Delhi & Chandigarh | 1905 | 1956 | 1956 | 1956 |
| Punjab | 2002 | 1941 | 2002 | 2002 |
| Himachal Pradesh | 1972 | 1972 | 1972 | 1972 |
| J & k | 1905 | 1954 | 1954 | 1954 |
| West Rajasthan | 2001 | 1983 | 1983 | 1983 |
| East Rajasthan | 1966 | 1966 | 1966 | 1966 |
| West Madhya Pradesh | 1962 | 1962 | 1978 | 1962 |
| East Madhya Pradesh | 1927 | 1932 | 1927 | 1827 |
| Gujarat Region | 2007 | 2007 | 1926 | 2007 |
| Saurashtra & Kutch | 2009 | 1979 | 2009 | 2009 |
| Kankan & Goa | 1926 | 1931 | 1931 | 1931 |
| Madhya Maharashtra | 1930 | 1930 | 1930 | 1930 |
| Marathwada | 1931 | 1931 | 1931 | 1931 |
| Vidarbha | 1968 | 1968 | 1968 | 1968 |
| Chhattisgarh | 1966 | 1966 | 1966 | 1966 |
| Coastal Andhra Pradesh | 2007 | 1915 | 2007 | 2007 |
| Telengana | 1924 | 1957 | 1957 | 1957 |
| Rayalseema | 1994 | 1979 | 1979 | 1979 |
| Tamilnadu | 2008 | 2008 | 1951 | 2008 |
| Coastal Karnataka | 1950 | 1950 | 1950 | 1950 |
| North Interior Karnataka | 1946 | 1946 | 1950 | 1946 |
| South Interior Karnataka | 1957 | 1957 | 1957 | 1957 |
| Kerala | 1967 | 1967 | 1967 | 1967 |

**Supplementary Table 2** Results of the slope value of innovative trend for annual and seasonal rainfall for the period of 1901–2015

| **Meteorological sub-divisions** | **Monsoon** | | **Post Monsoon** | **Summer** | **Winter** |
| --- | --- | --- | --- | --- | --- |
| Arunachal Pradesh | -4.404415 | | -7.264935 | -2.873567 | -1.979507 |
| Assam & Meghalaya | -0.8576076 | | -0.397386 | -1.674078 | -1.495376 |
| Nagaland, Manipur, Mizoram & Tripura | -1.720782 | | -1.72937 | -0.8784125 | -2.062478 |
| Sub Himalayan West Bengal & Sikkim | 0.0993395 | | 1.191207 | 1.908603 | 7.861272 |
| Gangetic West Bengal | 0.9903762 | | 0.9298703 | 0.05788661 | -1.861742 |
| Orissa | | -0.6459985 | -2.047875 | -0.7425854 | -2.475518 |
| Jharkhand | -1.131173 | | -0.3993101 | -1.089957 | -6.257645 |
| Bihar | -0.8325223 | | 2.283932 | 0.05230192 | -4.556307 |
| East Uttar Pradesh | -1.260413 | | 0.5238365 | -0.0247296 | -4.186432 |
| West Uttar Pradesh | -0.1145158 | | 0.665863 | 1.328757 | -3.891027 |
| Uttarakhand | -0.4532426 | | 0.06838508 | 5.058733 | -0.6010584 |
| Haryana, Delhi & Chandigarh | | 1.018007 | 0.7700277 | 3.84571 | -3.124029 |
| Punjab | 0.437545 | | -1.549639 | 1.019091 | -2.864704 |
| Himachal Pradesh | -0.6559786 | | 2.541323 | 2.603809 | 0.788809 |
| J & k | 1.399036 | | 3.327594 | 3.427299 | -0.7843613 |
| West Rajasthan | 0.3254377 | | 6.220131 | 5.880716 | -1.865162 |
| East Rajasthan | -0.5902886 | | 1.686459 | -1.10774 | -5.615679 |
| West Madhya Pradesh | -0.004757439 | | 1.137265 | -1.204317 | -4.139649 |
| East Madhya Pradesh | -1.455747 | | -1.768394 | -4.935095 | -4.300659 |
| Gujarat Region | 0.3396786 | | 1.50221 | -5.655325 | -10.94545 |
| Saurashtra& Kutch | 2.262771 | | 12.02951 | -5.569147 | -11.36608 |
| Kankan & Goa | 0.8823337 | | -0.7227555 | 1.35825 | -12.18585 |
| Madhya Maharashtra | 1.37044 | | 0.07092772 | -0.3750933 | -8.663061 |
| Marathwada | 0.2429673 | | 3.007552 | -0.5943488 | -7.664071 |
| Vidarbha | -0.5827009 | | 0.581144 | -2.9395 | -5.567284 |
| Chhattisgarh | -1.421429 | | -1.847206 | -4.97054 | -7.246132 |
| Coastal Andhra Pradesh | 0.5080996 | | -0.4162863 | -0.2732332 | -1.92337 |
| Telengana | 1.29264 | | 2.972832 | -0.871095 | -3.253601 |
| Rayalseema | 0.7595222 | | 1.294781 | -1.12729 | -7.584684 |
| Tamilnadu | -0.3588008 | | 0.340570 | -2.343954 | -6.887495 |
| Coastal Karnataka | 1.173413 | | 0.4249613 | 2.890478 | -8.017624 |
| Kerala | -0.8012287 | | -1.117401 | 0.76 | -4.379113 |

**Note:** The number in – negative sign indicates a decreasing trend.

**Supplementary Table 3** Meteorological division’s wise plotting of predicted and observed rainfall using ANN (Multilayer perceptron)

| **Sl No** | **Meteorological Sub Division** | **Predicted rainfall using ANN** |
| --- | --- | --- |
| 1 | **Arunachal Pradesh**  **↓** | 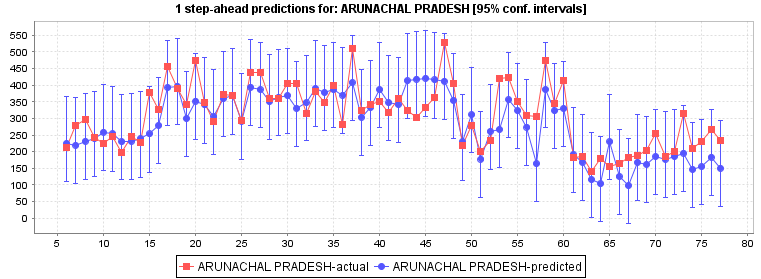 |
| 2 | **Assam**  **↓** | 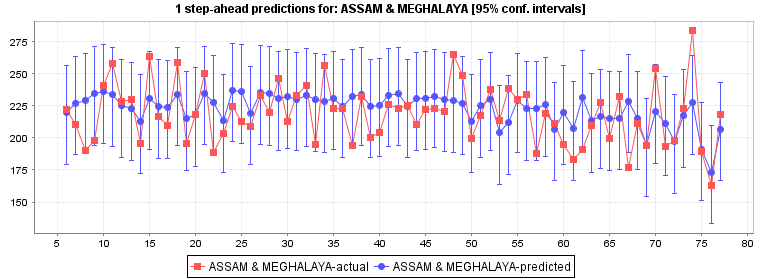 |
| 3 | **Naga Mani Mizo Tripura**  **↓** | 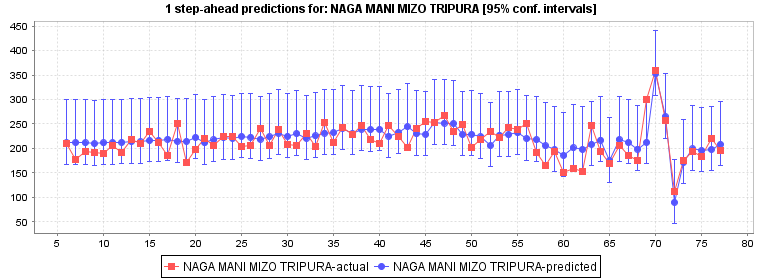 |
| 4 | **Sub Himalayan West Bengal & Sikkim**  **↓** | 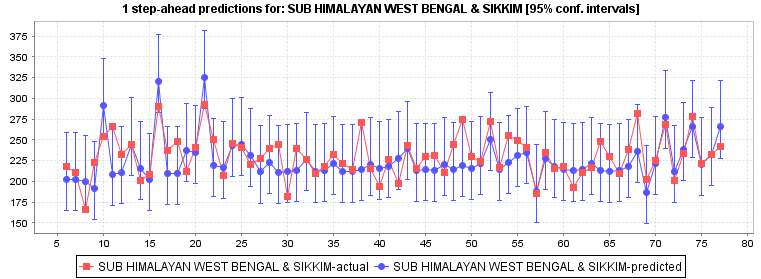 |
| 5 | **Gangetic West Bengal ↓** | 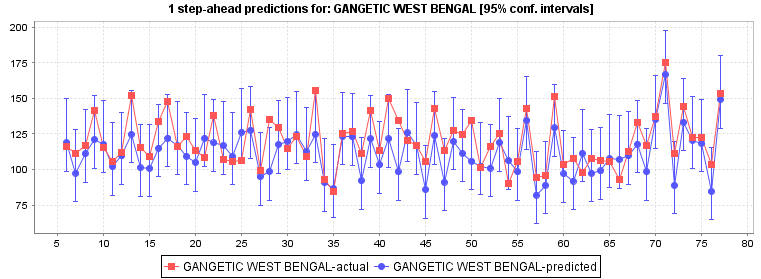 |
| 6 | **Orissa**  **↓** | 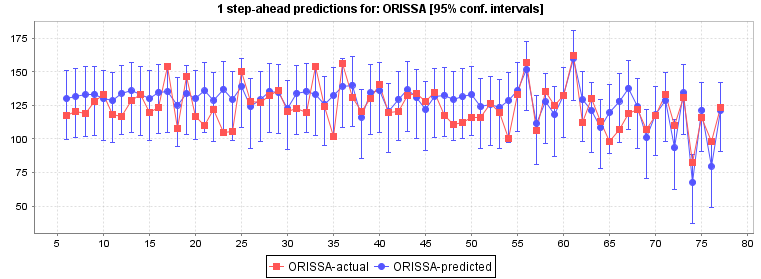 |
| 7 | **Jharkhand ↓** | 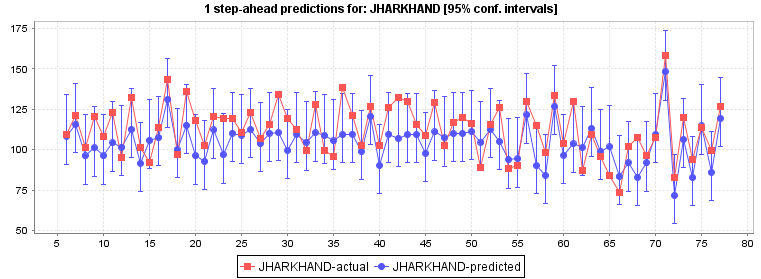 |
| 8 | **Bihar**  **↓** | 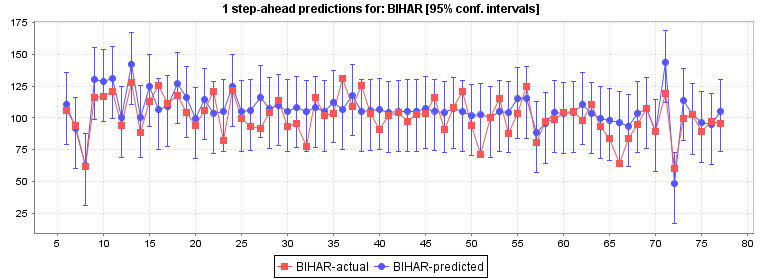 |
| 9 | **East Uttar Pradesh**  **↓** | 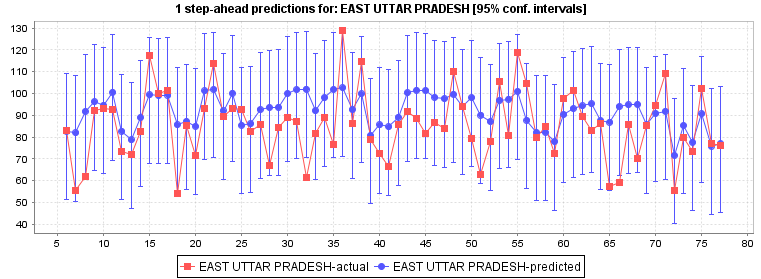 |
| 10 | **West Uttar Pradesh**  **↑** | 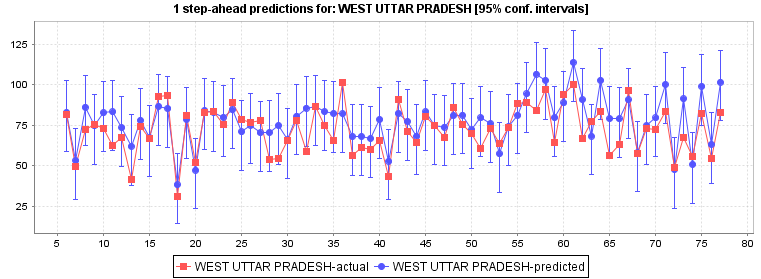 |
| 11 | **Uttarakhand ↓** | 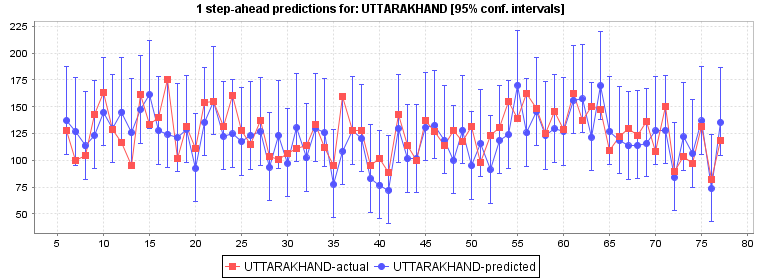 |
| 12 | **Haryana Delhi & Chandigarh ↑** | 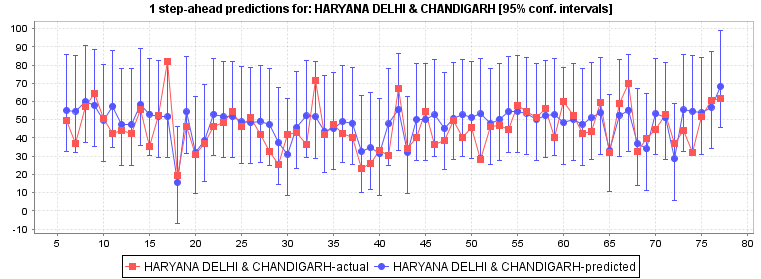 |
| 13 | **Punjab**  **↑** | 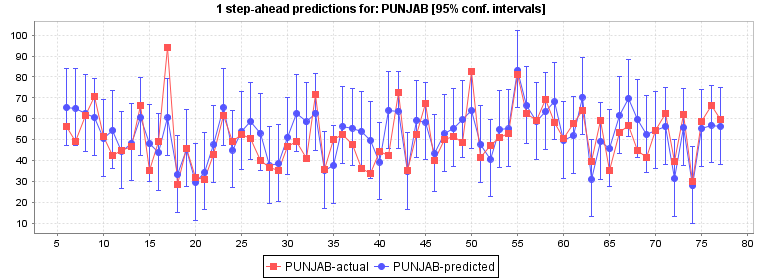 |
| 14 | **Himachal Pradesh**  **↓** | 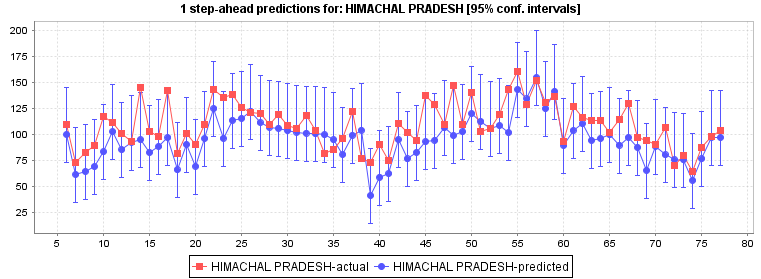 |
| 15 | **West Rajasthan ↑** | 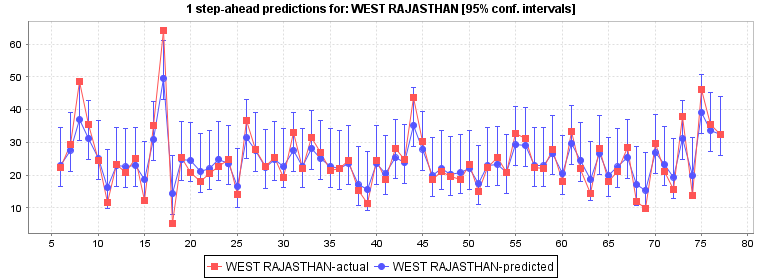 |
| 16 | **East Rajasthan ↑** | 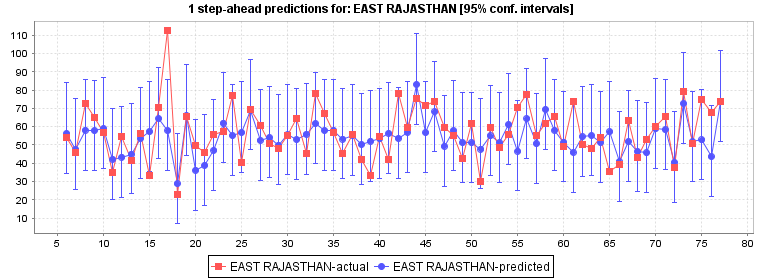 |
| 17 | **East Madhya Pradesh**  **↓** | 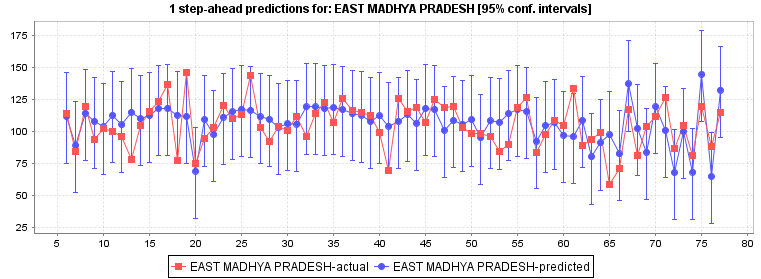 |
| 18 | **Gujarat region**  **↑** | 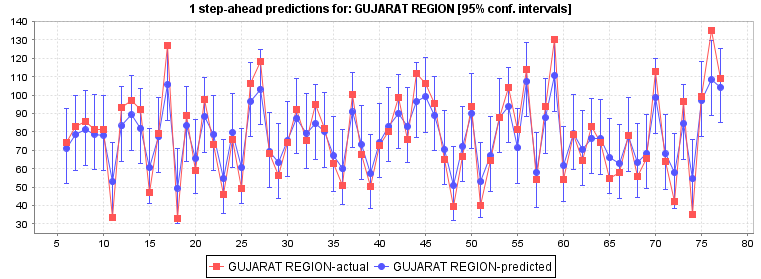 |
| 19 | **Saurashtra & Kutch**  **↑** | 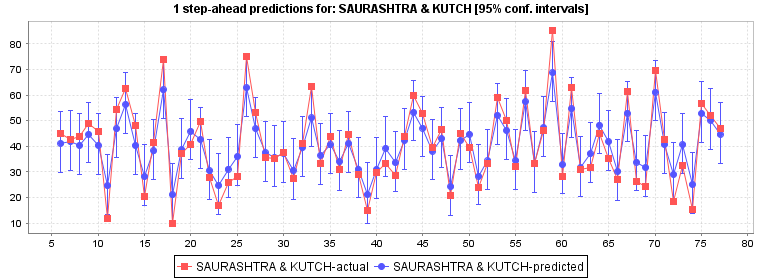 |
| 20 | **Konkan & Goa**  **↓** | 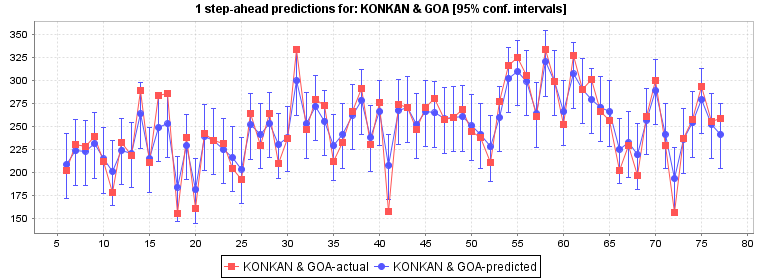 |
| 21 | **Madhya Maharashtra ↑** | 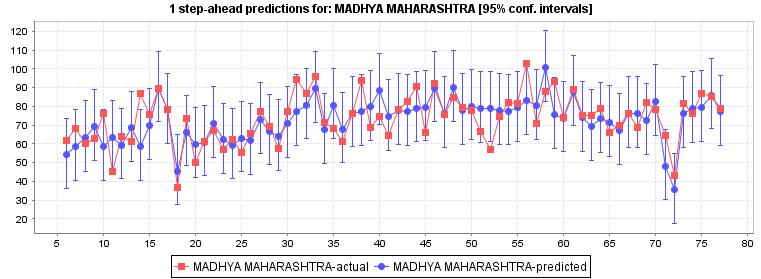 |
| 22 | **Matathwada ↑** | 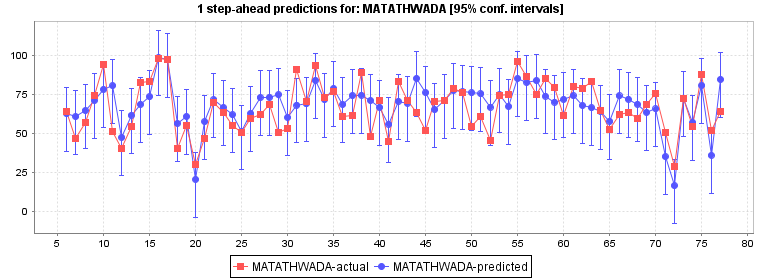 |
| 23 | **Vidarbha**  **↓** | 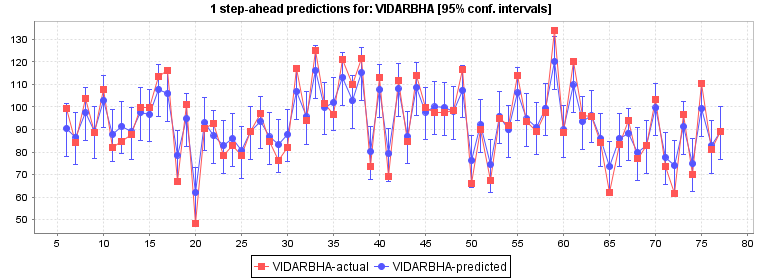 |
| 24 | **Chhattisgarh**  **↓** | 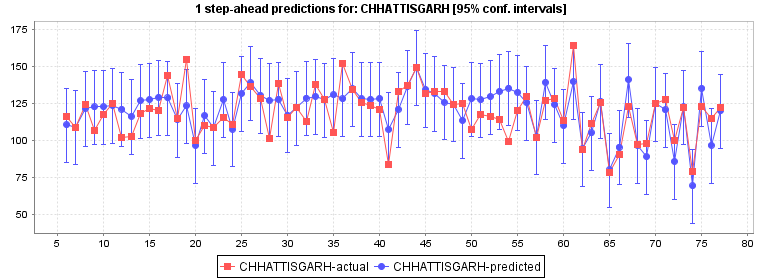 |
| 25 | **Coastal Andhra Pradesh**  **↑** | 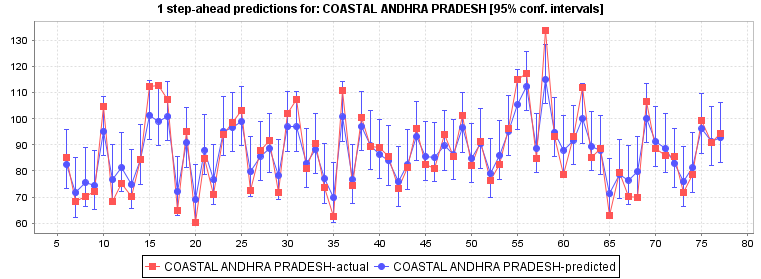 |
| 26 | **Telangana ↑** | 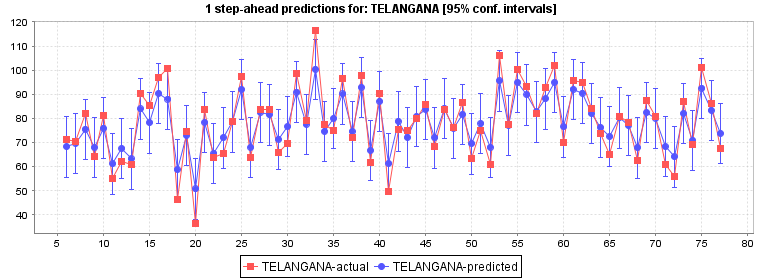 |
| 27 | **Rayalseema ↑** | 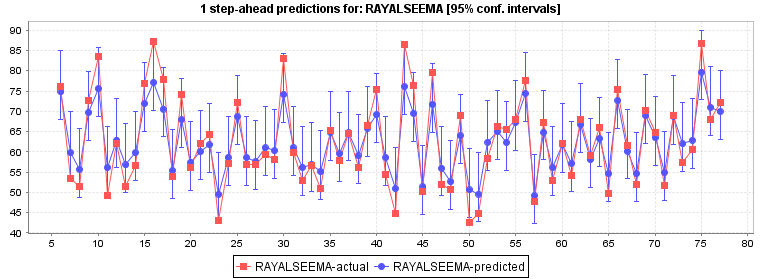 |
| 28 | **Tamil nadu ↑** | 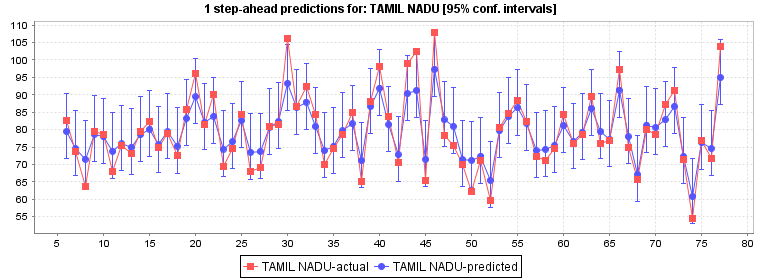 |
| 29 | **Coastal Karnataka ↓** | 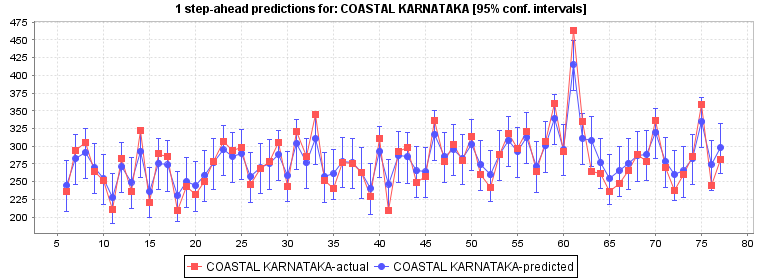 |
| 30 | **North interior Karnataka ↑** | 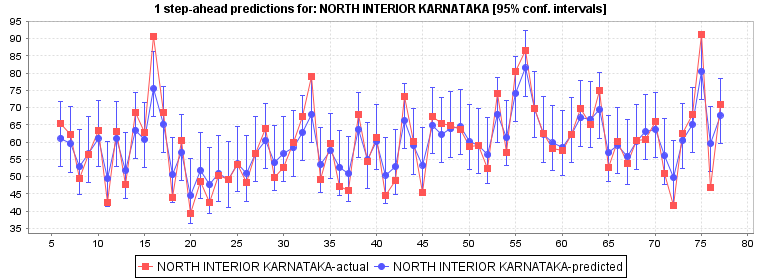 |
| 31 | **South interior Karnataka ↑** | 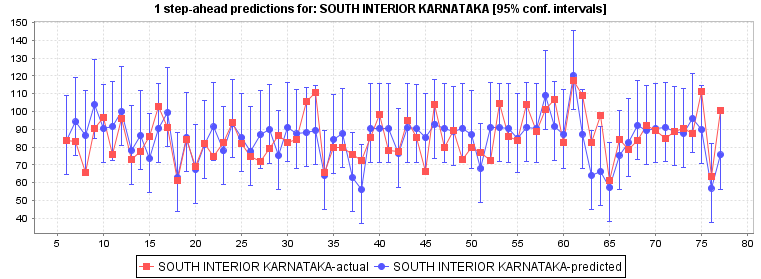 |
| 32 | **Kerala**  **↓** | 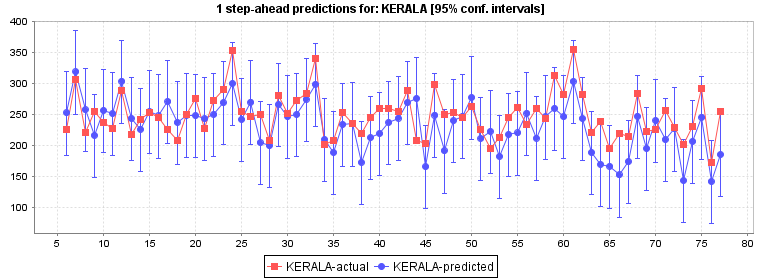 |
| 33 | **West Madhya**  **Pradesh**  **↓** | 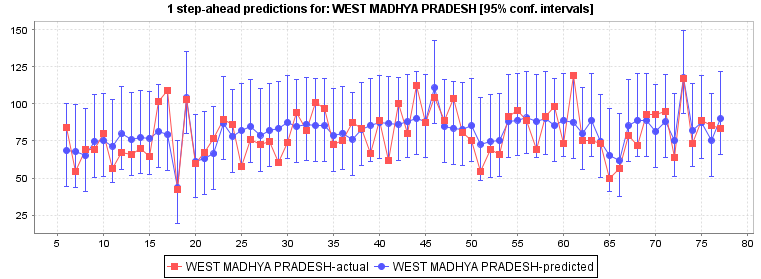 |
| 34 | **Jammu and Kashmir**  **↑** | 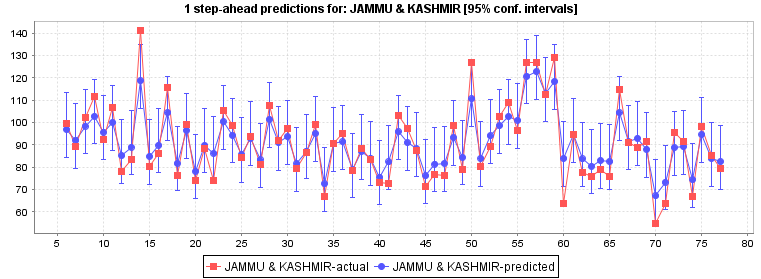 |

N.B.- ↑ represent upward trend of rainfall and ↓ represent downward trend of rainfall

**Supplementary Table 4** Meteorological sub-division wise 15 incoming years rainfall forecasting

| **Sl No** | **Meteorological Sub Division** | **ANN Forecasting** | **RMSE, MAE** |
| --- | --- | --- | --- |
| 1 | **Arunachal Pradesh**  **↓** | 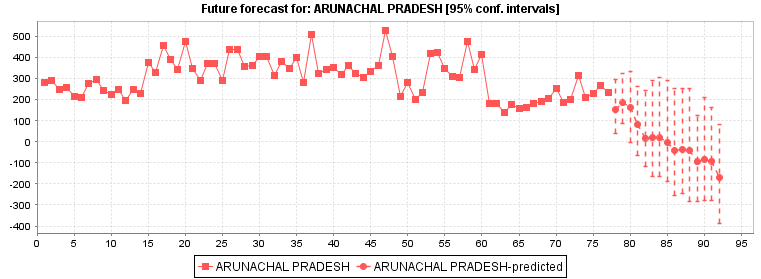 | 47.34, 60.65 |
| 2 | **Assam & Meghalaya**  **↓** | 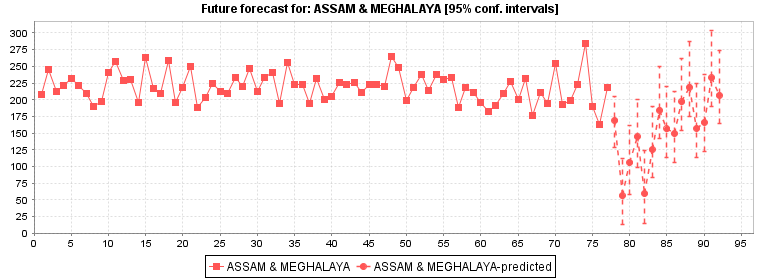 | 16.14, 20.71 |
| 3 | **Naga, Mani, Mizo & Tripura**  **↓** | 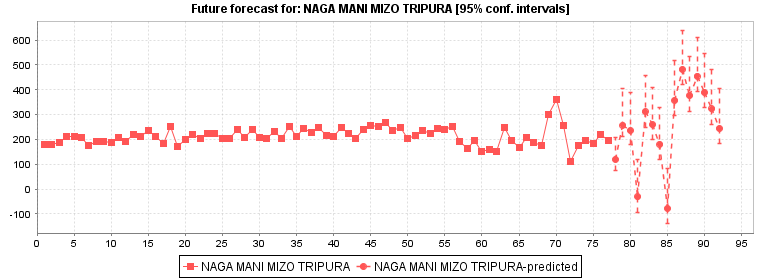 | 18.91, 23.80 |
| 4 | **Sub Himalayan West Bengal & Sikkim**  **↓** | 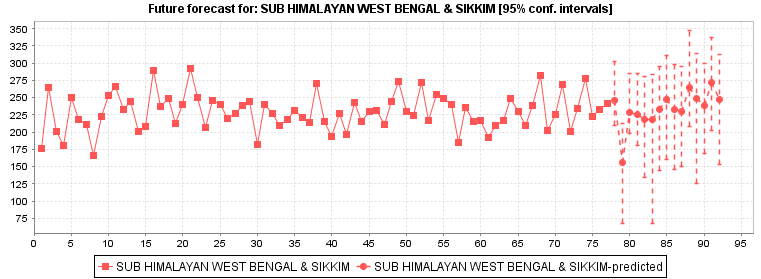 | 16.66, 21.93 |
| 5 | **Gangetic West Bengal**  **↓** | 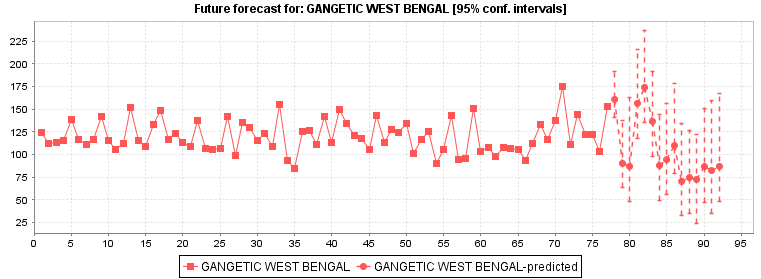 | 11.95, 14.91 |
| 6 | **Orissa**  **↓** | 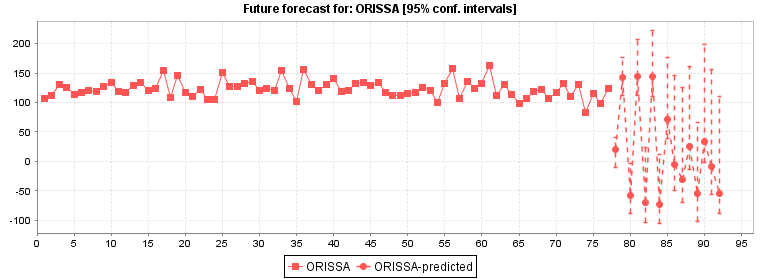 | 10.17, 12.87 |
| 7 | **Jharkhand**  **↓** | 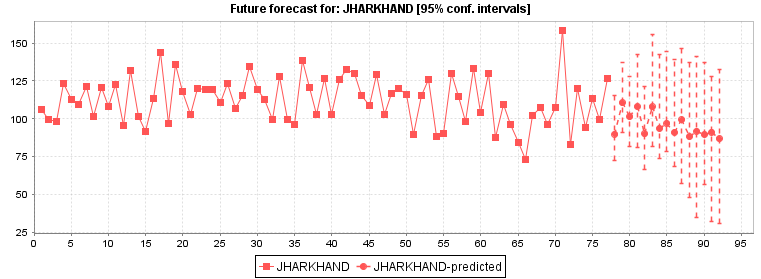 | 11.34, 13.40 |
| 8 | **Bihar**  **↓** | 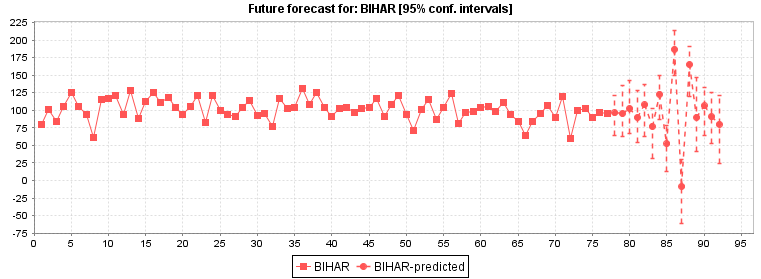 | 9.80, 12.40 |
| 9 | **East Uttar Pradesh**  **↓** | 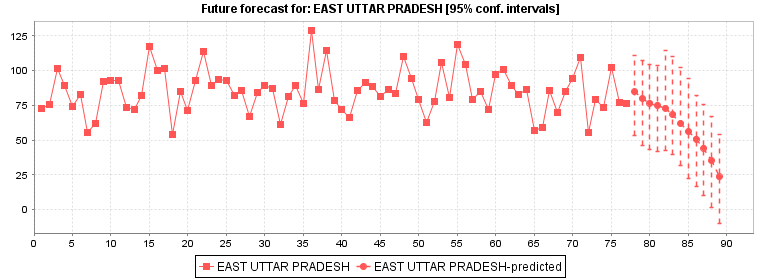 | 11.58, 14.85 |
| 10 | **West Uttar Pradesh**  **↑** | 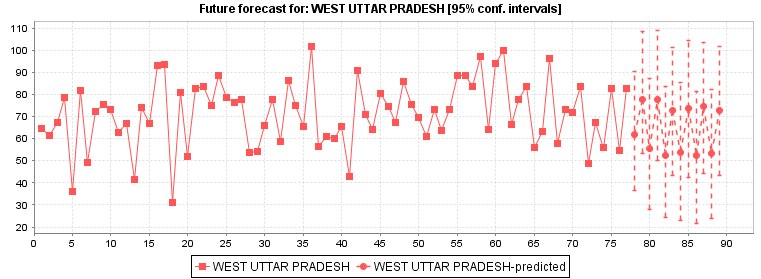 | 9.83, 13.56 |
| 11 | **Uttarakhand**  **↓** | 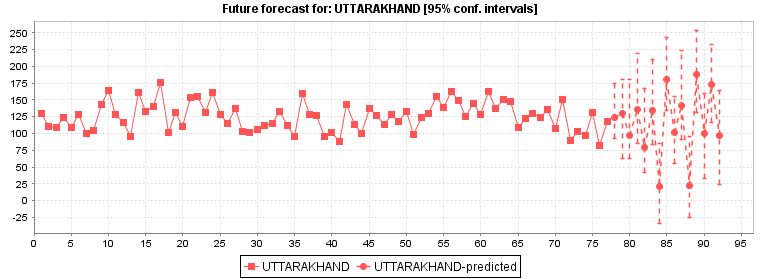 | 16.07, 19.58 |
| 12 | **Haryana, Delhi & Chandigarh**  **↑** | 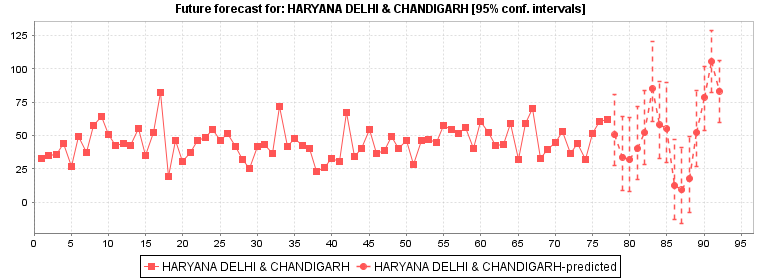 | 7.49, 9.79 |
| 13 | **Punjab**  **↑** | 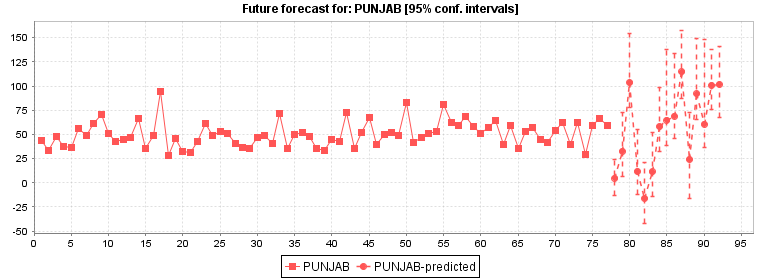 | 7.24, 9.36 |
| 14 | **Himachal Pradesh**  **↓** | 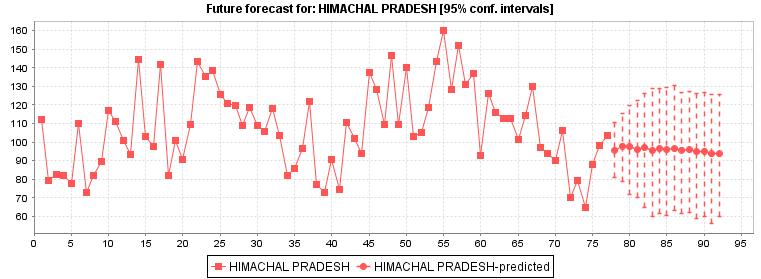 | 16.02, 20.27 |
| 15 | **J & K**  **↑** | 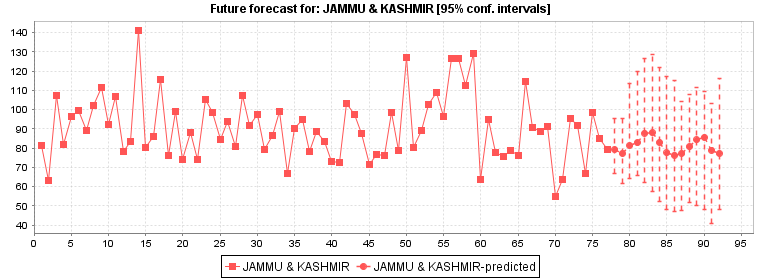 | 5.15, 6.66 |
| 16 | **West Rajasthan**  **↑** | 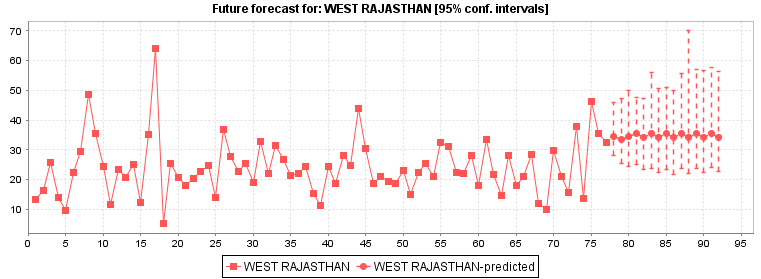 | 2.84, 3.89 |
| 17 | **East Rajasthan**  **↑** | 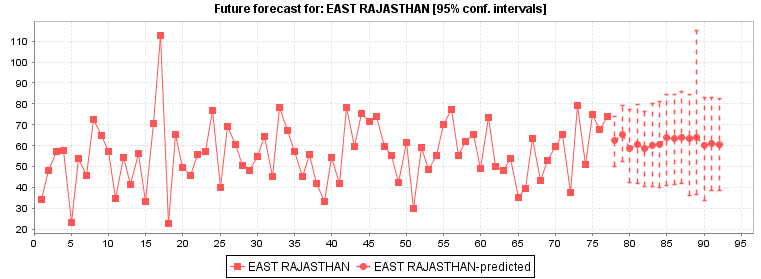 | 9.12, 12.75 |
| 18 | **West Madhya**  **Pradesh**  **↓** | 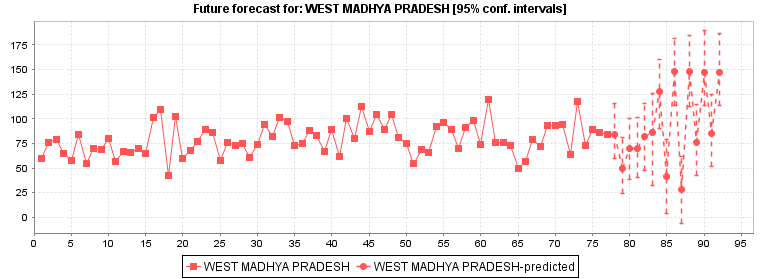 | 9.87, 12.24 |
| 19 | **East Madhya Pradesh**  **↓** | 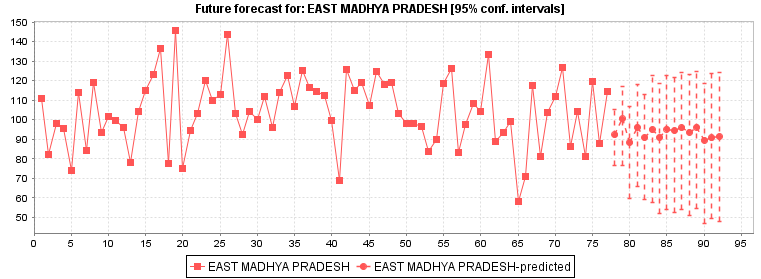 | 5.20, 6.77 |
| 20 | **Gujarat Region**  **↑** | 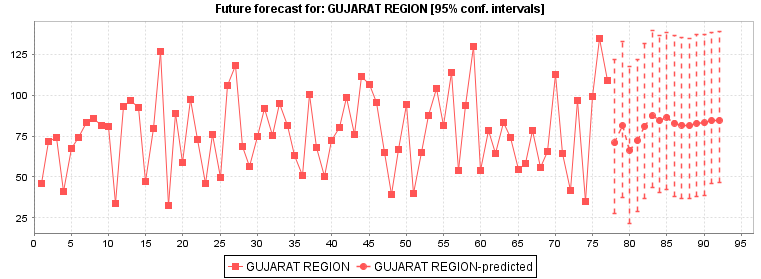 | 7.69, 9.44 |
| 21 | **Saurashtra & Kutch**  **↑** | 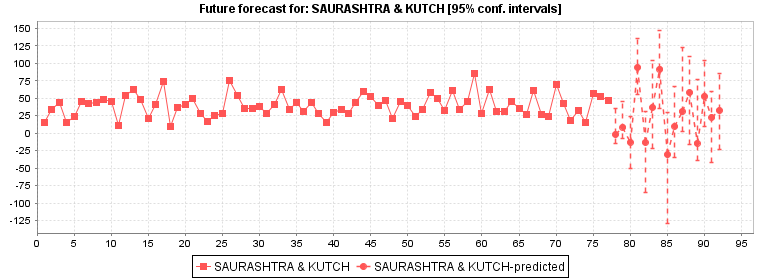 | 5.11, 6.97 |
| 22 | **Konkan & Goa**  **↓** | 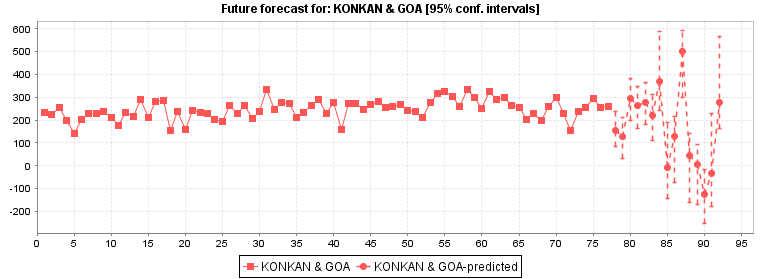 | 11.68, 15.41 |
| 23 | **Madhya Maharashtra**  **↑** | 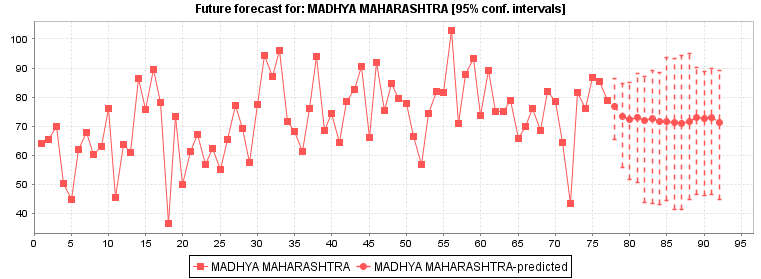 | 7.17, 9.26 |
| 24 | **Matathwada**  **↑** | 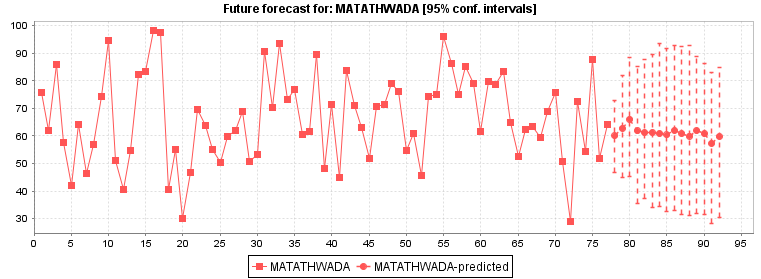 | 9.33, 11.68 |
| 25 | **Vidarbha**  **↓** | 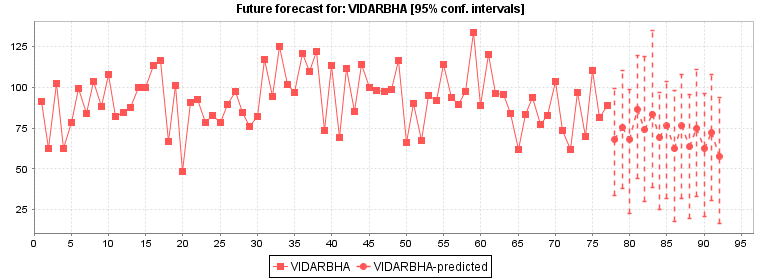 | 11.66, 14.22 |
| 26 | **Chhattisgarh**  **↓** | 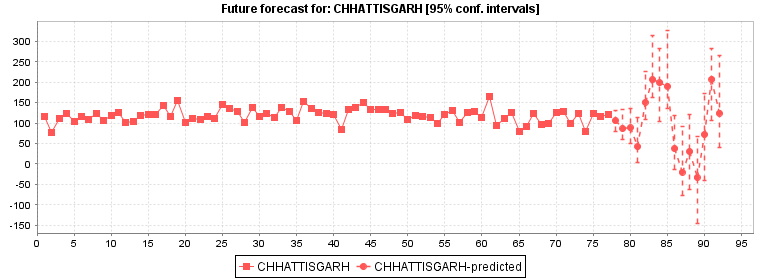 | 8.79, 12.13 |
| 27 | **Coastal Andhra Pradesh**  **↑** | 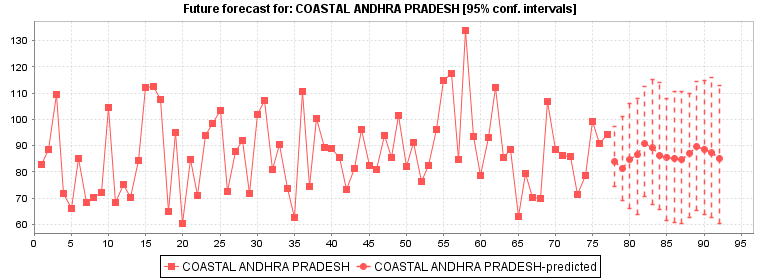 | 4.61, 5.80 |
| 28 | **Telangana**  **↑** | 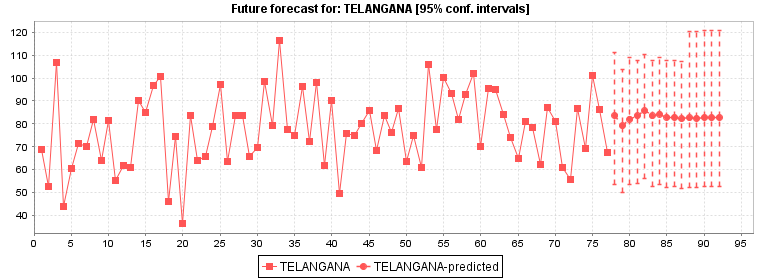 | 11.27, 14.42 |
| 29 | **Rayalseema**  **↑** | 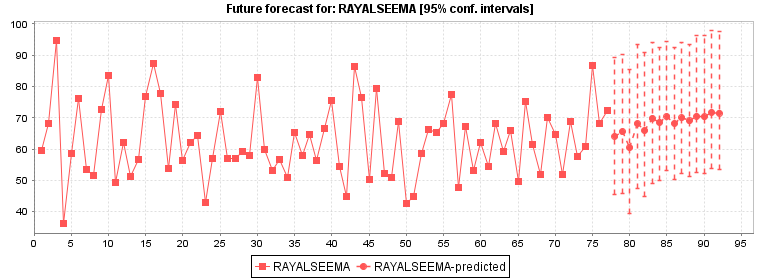 | 7.92, 10.53 |
| 30 | **Tamilnadu**  **↑** | 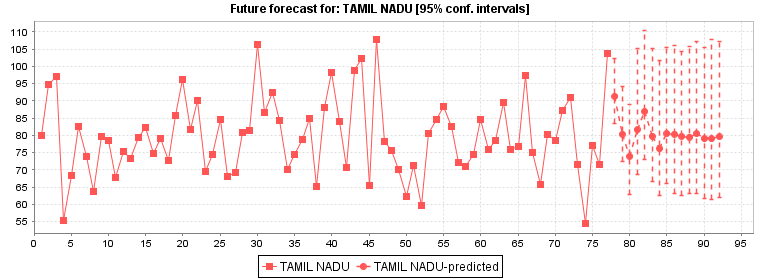 | 3.34, 4.41 |
| 31 | **Coastal Karnataka**  **↓** | 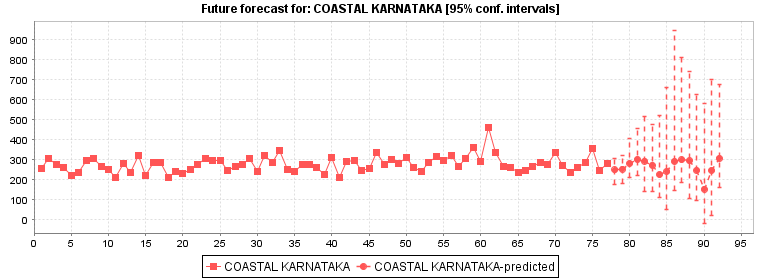 | 12.98, 16.37 |
| 32 | **North Interior Karnataka**  **↑** | 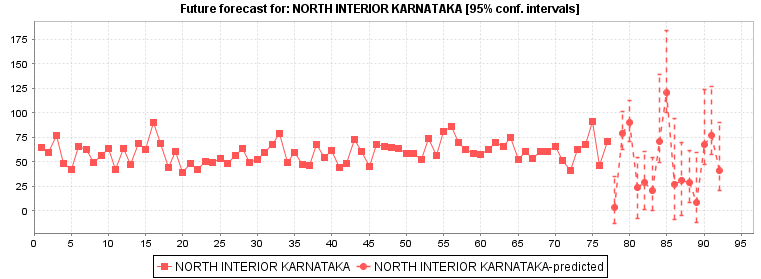 | 3.63, 4.70 |
| 33 | **South Interior Karnataka**  **↑** | 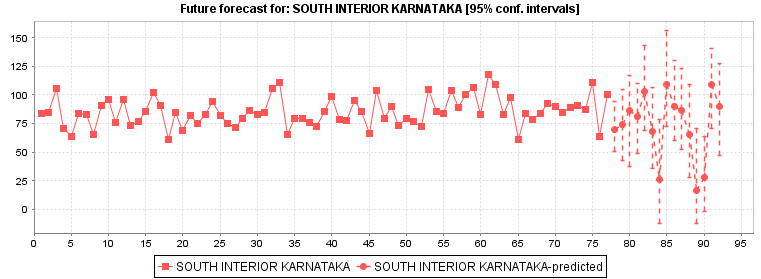 | 8.99, 11.34 |
| 34 | **Kerala**  **↓** | 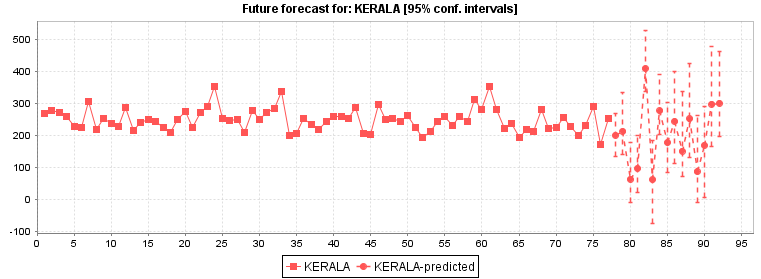 | 11.37, 14.41 |

N.B.- ↑ represent upward trend of rainfall and ↓ represent downward trend of rainfall

**List of Supplementary Figures**

**
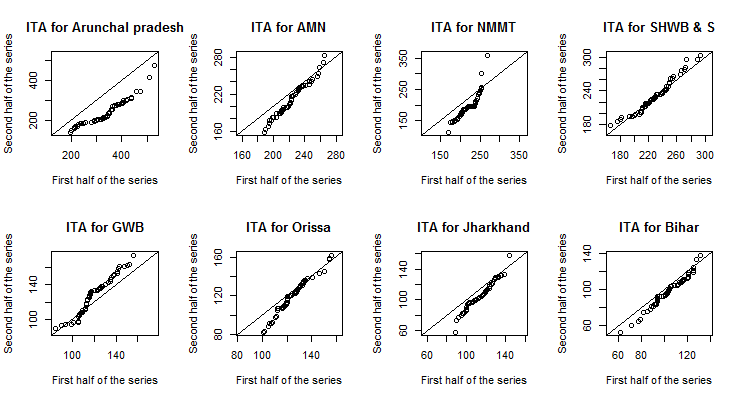
**

**
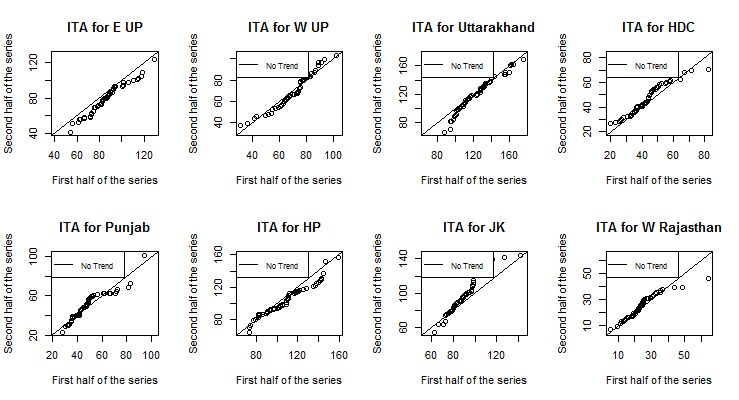
**

**Supplementary Figure 1 State wise innovative trend analysis of monsoon rainfall (Continued)**

**
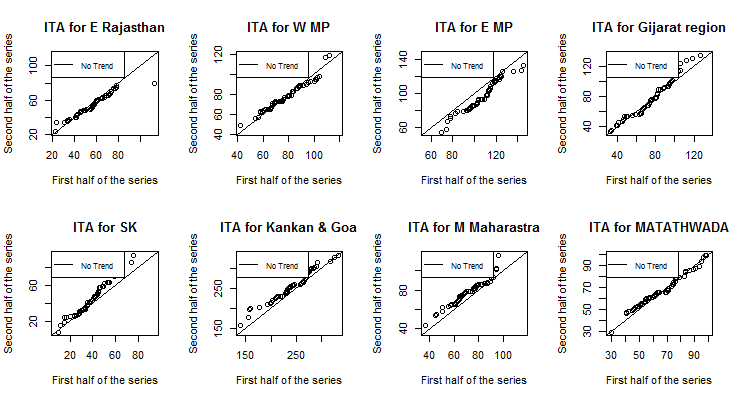
**

**
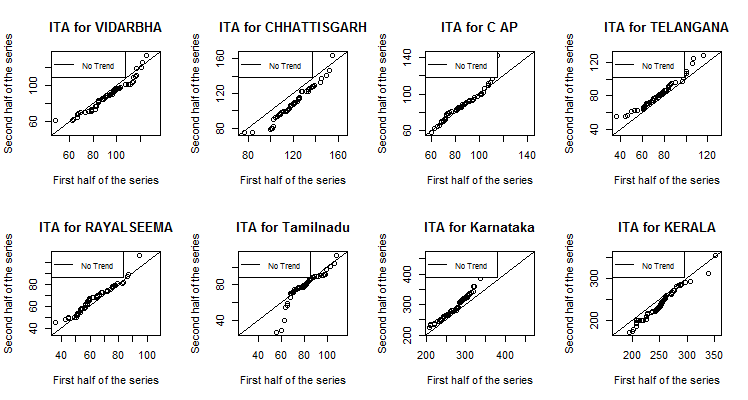
**

**Supplementary Figure 1 State wise innovative trend analysis of monsoon rainfall**

**
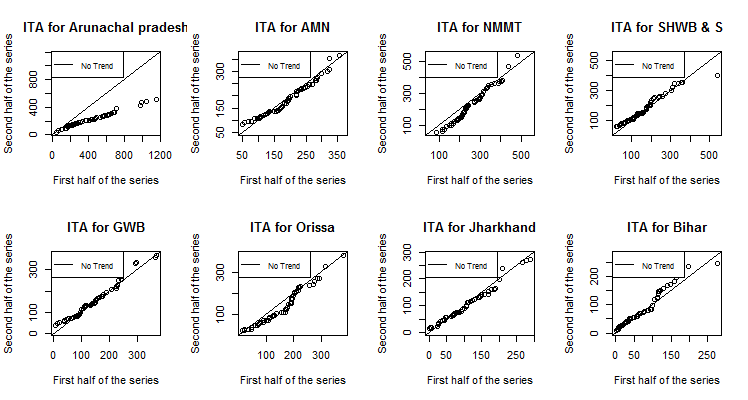
**

**
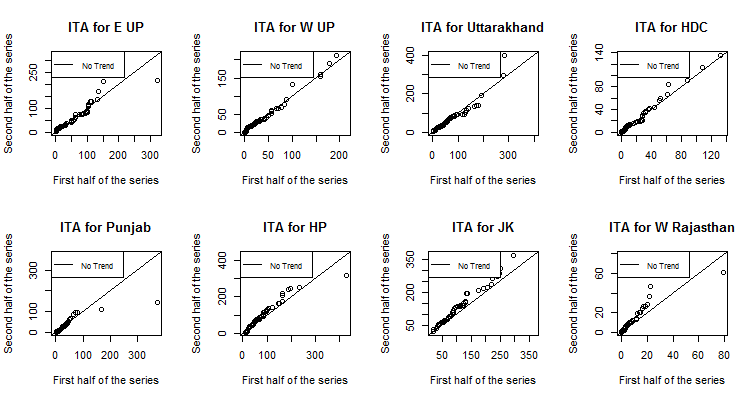
**

**Supplementary Figure 2 State wise innovative trend analysis of post monsoon rainfall (Continued)**

**
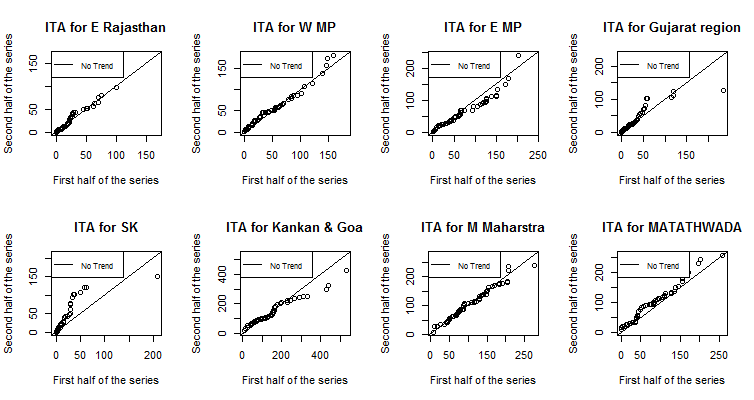
**

**
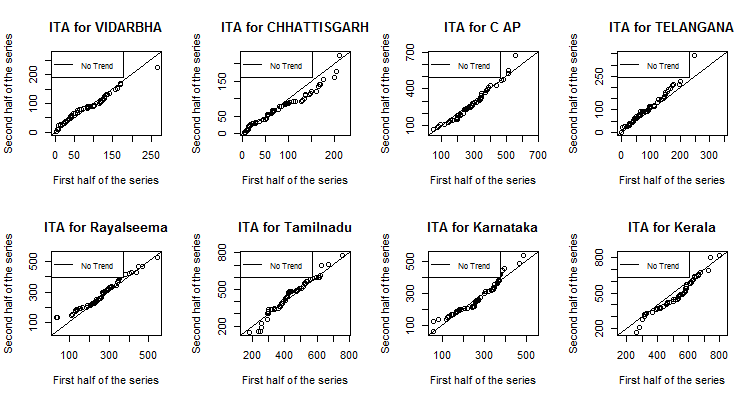
**

**Supplementary Figure 2 State wise innovative trend analysis of post monsoon rainfall**

**
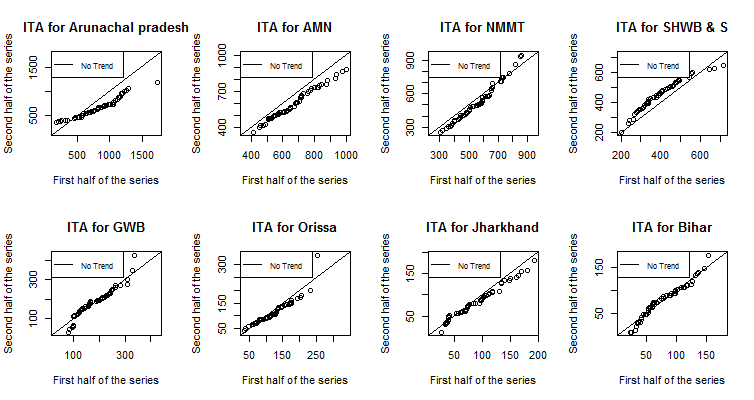
**

**
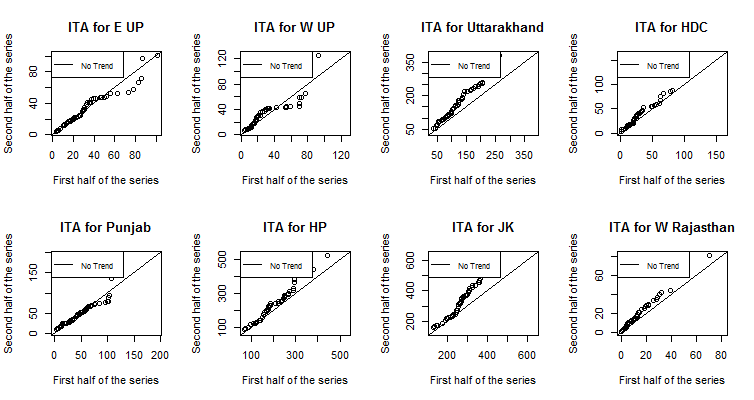
**

**Supplementary Figure 3 State wise innovative trend analysis of summer rainfall (continued)**

**
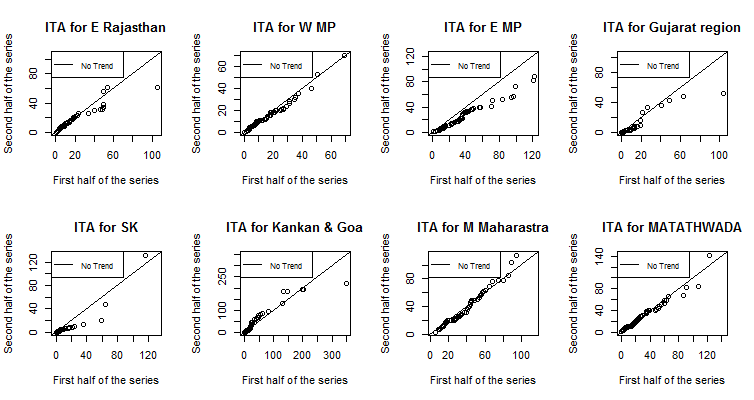
**

**
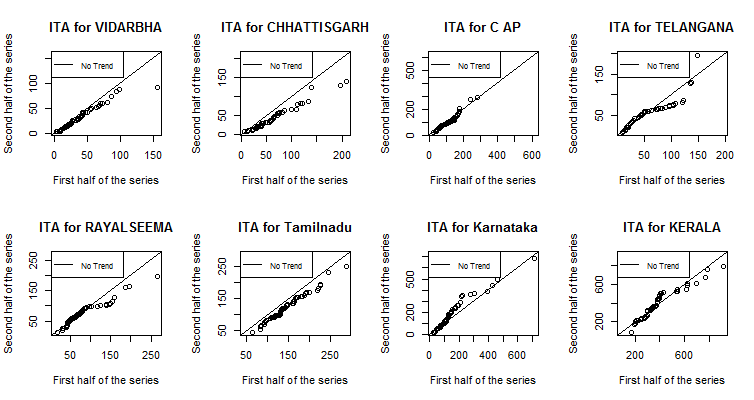
**

**Supplementary Figure 3 State wise innovative trend analysis of summer rainfall**

**
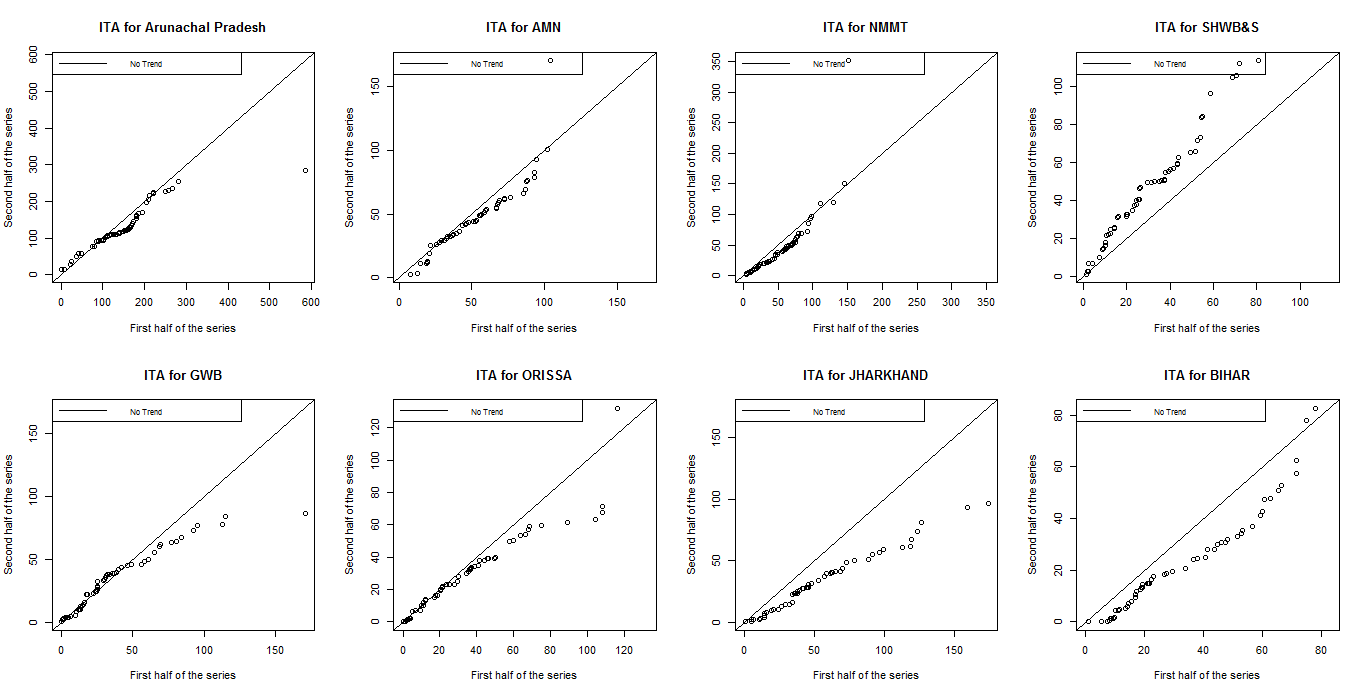
**

**
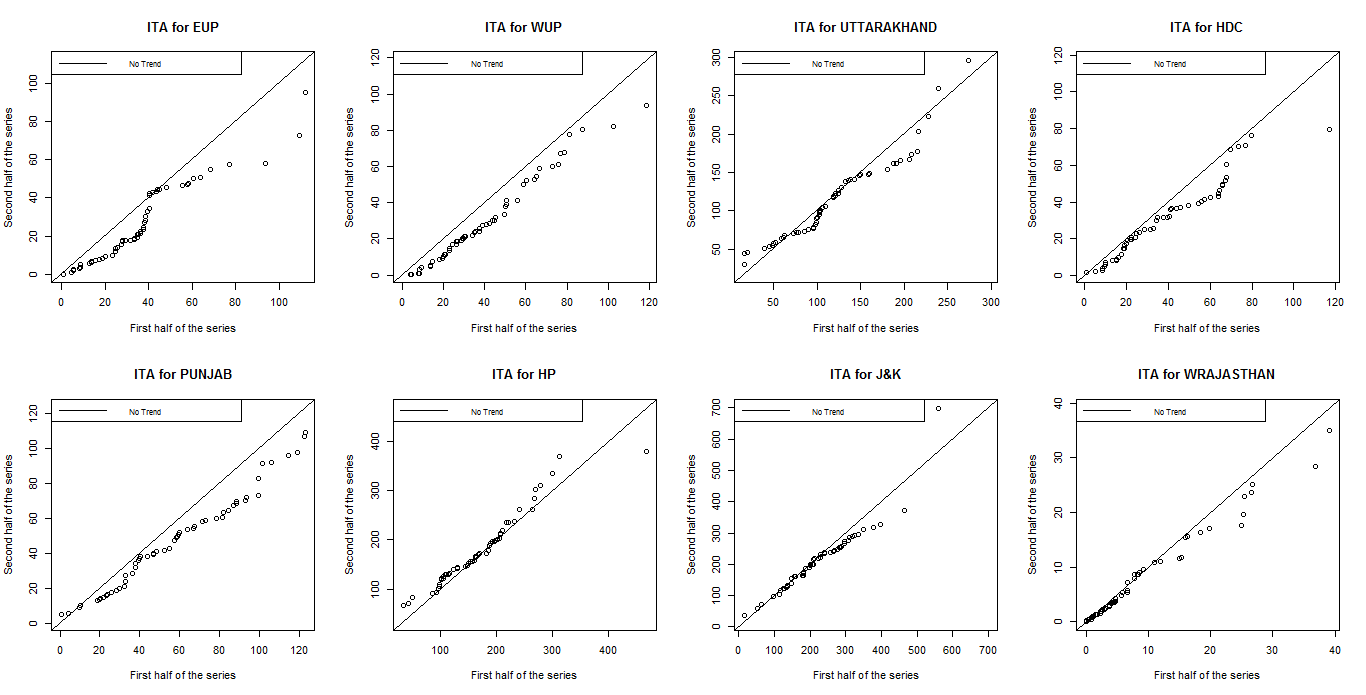
**

**Supplementary Figure 4 State wise innovative trend analysis of winter rainfall (Continued)**

**
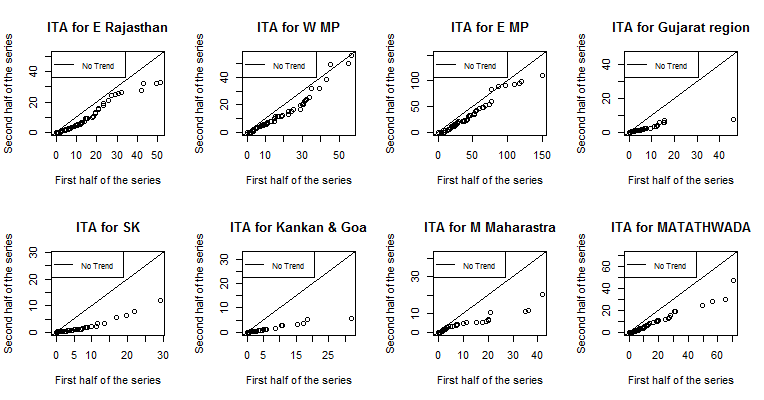
**

**
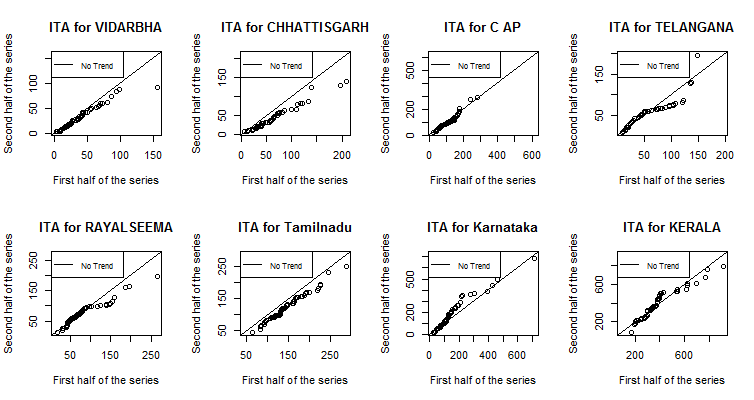
**

**Supplementary Figure 4 State wise innovative trend analysis of winter rainfall**
